# p16^Ink4a^‐Positive Hepatocytes Drive Liver Fibrosis Through Activation of LIFR Family Pathway

**DOI:** 10.1002/advs.202510562

**Published:** 2026-01-25

**Authors:** Koji Nishikawa, Teh‐Wei Wang, Satoshi Kawakami, Shota Tanimoto, Kiyoshi Yamaguchi, Taketomo Kido, Masamichi Kimura, Tsunekazu Hishima, Yuki T. Okamura, Satotaka Omori, Takumi Iritani, Toshikaze Chiba, Takehiro Jimbo, Michio Katano, Kansuporn Kamataki, Ryoichi Yokoyama, Eigo Shimizu, Kiminori Kimura, Satoshi Yamzaki, Seiya Imoto, Yoichi Furukawa, Atsushi Miyajima, Yoshikazu Johmura, Makoto Nakanishi

**Affiliations:** ^1^ Division of Cancer Cell Biology The Institute of Medical Science The University of Tokyo Tokyo Japan; ^2^ Department of Hepatology Tokyo Metropolitan Cancer and Infectious Diseases Center Komagome Hospital Tokyo Japan; ^3^ Project Division of Generative AI Utilization Aging Cells The Institute of Medical Science The University of Tokyo Tokyo Japan; ^4^ Division of Clinical Genome Research The Institute of Medical Science The University of Tokyo Tokyo Japan; ^5^ Laboratory of Cell Growth and Differentiation Institute for Quantitative Biosciences The University of Tokyo Tokyo Japan; ^6^ Department of Pathology Tokyo Metropolitan Cancer and Infectious Diseases Center Komagome Hospital Tokyo Japan; ^7^ Integrated Biosciences Redwood City California USA; ^8^ GMO Internet Group, Inc. Tokyo Japan; ^9^ GMO Research Activity Support & Technology, Inc. Tokyo Japan; ^10^ Division of Health Medical Intelligence Human Genome Center Center for Experimental Medicine and Systems Biology The Institute of Medical Science The University of Tokyo Tokyo Japan; ^11^ Division of Cell Regulation Center of Experimental Medicine and Systems Biology The Institute of Medical Science The University of Tokyo Tokyo Japan; ^12^ Division of Cancer and Senescence Biology Cancer Research Institute Kanazawa University Kakuma Kanazawa Japan

**Keywords:** cellular senescence, LIFR pathway, liver fibrosis

## Abstract

As organs undergo the process of aging, they exhibit signs of progressive fibrosis, a hallmark of aging that is observed in various organs, including the liver, kidneys, and lungs. Liver fibrosis is a particularly deleterious outcome of the healing processes that occur during the repair of chronic liver injury. It is widely accepted that the majority of these injuries are initially triggered by hepatocytes. Indeed, elderly patients have been shown to be more prone to developing liver fibrosis following hepatic injury. However, the mechanisms by which aging promotes fibrotic processes remain to be elucidated. The preceding observation, indicating a robust correlation between the severity of fibrosis in human cirrhotic patients and the population of hepatocytes expressing elevated levels of p16^Ink4a^ (p16^h^), proposes that p16^h^ hepatocytes might serve as initiators of fibrogenic processes in response to liver injury. In this study, we employed a CCl_4_‐induced hepatitis model to promote a fibrogenic process and observed the accumulation of p16^h^ hepatocytes in zone 3. These p16^h^ cells manifest numerous senescent characteristics, and their accumulation has been strongly correlated with the severity of liver fibrosis. Selective elimination of p16^h^ hepatocytes has been shown to ameliorate CCl_4_‐induced liver fibrosis, presumably through the suppression of hepatic stellate cell activation. Single‐cell transcriptomic analysis revealed that murine and human hepatocytes up‐regulated Ctf1 or Lif, the ligands of the LIFR signaling pathway. The administration of LIFR ligands has been demonstrated to enhance the phosphorylation of STAT3, and the LIFR inhibitor rescued the fibrogenic phenotype in hepatic stellate cells induced by secreted factors from senescent hepatocytes. This finding offers potential therapeutic insights for the management of liver fibrosis.

## Graphical abstract

This study found that, following the long‐term CCl4 treatment, p16high hepatocytes appeared in zone 3, spatially co‐localizing with fibrotic areas. A specific cluster of p16^high^ hepatocytes upregulated CTF1/LIF expression which induced HSC activation and further liver fibrosis, as revealed by single cell transcriptomic analysis. The transcriptomic signatures of mouse p16^high^ hepatocytes exhibited a significantly enrichment in a subset of human hepatocytes derived from liver cirrhotic patients, as opposed to those from healthy donors.

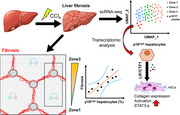

## 1 Introduction

Aging is associated with an increase in chronic inflammation, which is a significant contributing factor to fibrosis in various organs. Approximately one‐third of disease‐related deaths are due to organ fibrosis and its associated functional failure [1, 2]. Liver fibrosis is an aberrant repair response to a variety of chronic liver injuries caused by viral infection, metabolic dysfunction, and autoimmune response. It is characterized by excessive deposition of diffuse extracellular matrix (ECM) with replacement of hepatic parenchyma and abnormal connective tissue hyperplasia. From a clinical perspective, elderly patients demonstrate a heightened propensity to manifest severe fibrosis in the context of chronic liver diseases, such as nonalcoholic fatty liver disease (NAFLD) or hepatitis C. These patients often exhibit a more expeditious progression to advanced fibrosis and cirrhosis in comparison to their younger counterparts [3, 4, 5]. Liver fibrosis and subsequent cirrhosis are believed to be an irreversible process, but recent studies have suggested that the fibrogenic process is reversible when the causative factors are removed [6]. Therefore, identification of the cells responsible for initiating fibrogenic processes and characterization of their signaling are prerequisites for establishing innovative therapeutic strategies to ameliorate fibrogenic pathogenesis. In addition, a better understanding of the mechanisms underlying the regression of fibrosis is also important.

Hepatic stellate cells (HSCs) are cells primarily responsible for the development of liver fibrosis. They serve as a storage site for retinyl esters and maintenance of liver homeostasis and normally stay in a quiescent state under unperturbed physiological conditions [7, 8]. Chronic injury to hepatocytes generates a variety of signals that stimulate the specific transcription factors and morphogens in quiescent HSCs, leading to their functional activation with the acquisition of pro‐fibrogenic and pro‐inflammatory properties [9]. Thus, the majority of the research has been focused on HSCs and their inflammatory pathways to understand the fibrogenic processes. However, the crosstalk between hepatocytes as the origin of injury cells and multiple signaling pathways to activate HSCs should not be overlooked. Future studies leading to a complete understanding of the specific hepatocytes and molecular signaling that activate HSCs will definitely give rise to new therapeutic strategies to treat liver fibrosis.

Cellular senescence is a fundamental and complex process as a protective mechanism against various cellular insults, such as DNA damage and oncogene activation, across all species. Senescence induction not only attenuates the proliferative potential of damaged cells and protects them from their malignant transformation, but also alters the tissue microenvironment and homeostasis through the secretion of various proinflammatory cytokines, chemokines, and growth factors (Senescent Associated Secretory Phenotypes: SASP), resulting in chronic inflammation [10]. A series of recent evidence has suggested that senescence of hepatocytes, cholangiocytes, HSCs, and immune‐related cells is involved in the progression of chronic liver disease [11]. However, recent genetic tracing, ablation, and manipulation of defined p16^Ink4a^‐positive (p16^Ink4a^+) cells, one of the most widely used senescent makers, although no single marker is sufficient to unequivocally identify a senescent cell, revealed that senescent cells in vivo exhibit heterogeneous properties, and senescence in different cell types have different pathophysiological functions in age‐related diseases is largely unknown [12]. For example, it has recently been reported that selective removal of p16^Ink4a^+ macrophages markedly attenuated CCl_4_‐induced liver injury, whereas removal of p16^Ink4a^+ endothelial cells exacerbated it, although the efficiency and duration of p16^Ink4a^+ cell removal may influence the pathological outcomes [13]. In this report, p16^Ink4a^+ hepatocytes were barely detected in CCl_4_‐induced liver injury and consequent liver fibrosis. However, in human liver samples from patients with non‐alcoholic fatty liver disease (NAFLD), p16^Ink4a^+ hepatocytes were predominantly detected, and there is a strong correlation between the proportion of p16^Ink4a^+ hepatocytes and the degree of liver fibrosis [14]. Taken together with the possibility that hepatocytes may initiate the signaling of fibrogenic processes in the liver, p16^Ink4a^‐expressing hepatocytes may play a specific role in the initiation of liver fibrosis.

In this study, we demonstrated the accumulation of hepatocytes highly expressing p16 (p16^h^ hepatocytes, which represent cells able to be labeled in p16‐Cre^ERT2^ model) in CCl_4_‐induced liver fibrosis. We also generated a Cre and Dre recombinase‐mediated genetic mouse model that allows specific manipulation of p16^h^ hepatocytes. Finally, we identified the LIFR family of signaling pathways as a factor linking p16^h^ hepatocytes to fibrogenic activation of hepatic stellate cells.

## 2 Results

### 2.1 p16^h^ Hepatocytes are Enriched in the Liver Fibrosis Model

**FIGURE 1:**
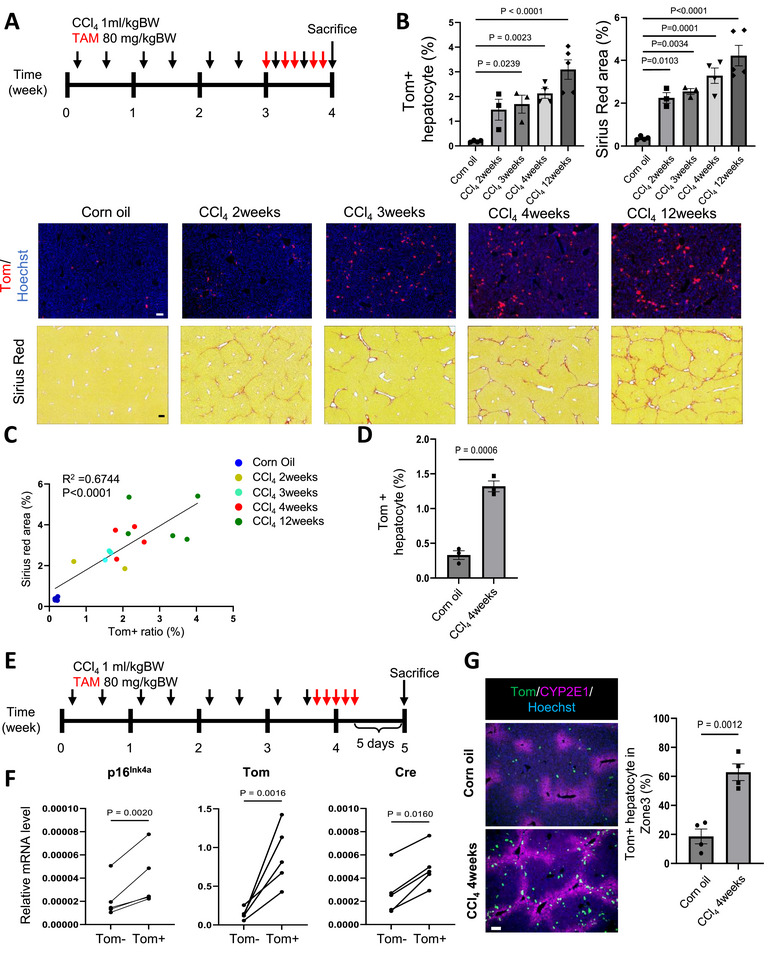
The p16^h^ hepatocytes are enriched in the CCl_4_‐induced liver fibrosis model (A) The schematic image of CCl_4_ and TAM administration. (B) Fluorescent imaging and Sirius red staining results of the liver treated with CCl_4_ at the indicated periods. Corn oil group represents the mice treated with vehicle for 4 weeks. The CCl_4_ and TAM injection frequency was shown as in (A) with indicated time periods. The representative images were shown in the lower panels. Scale bar, 100 µm. Quantitative results of Tom+ hepatocyte population and Sirius red area percentage were shown in the upper panel (n= 4, 3, 3, 4, 5 for each group). (C) Scatter plot of Sirius red area and Tom+ hepatocyte population shown in (B). Pearson correlation was shown in the figure. (D) FACS quantitative result of Tom+ hepatocyte percentage of livers treated as in (A) (n=3). (E) The schematic image of CCl_4_ and TAM administration for mRNA analysis of Tom+ and Tom‐ hepatocytes. (F) qPCR analysis of indicated genes in isolated hepatocytes (n=5). (G) Representative immunostaining images of livers treated as in (A). The tdTomato proteins were stained by anti‐mCherry. Scale, 100 µm. The percentage of Tom+ hepatocytes located in zone 3 was calculated and shown in the left panel (n=4). One‐way ANOVA followed by Dunnett's test was performed in (B). An unpaired *T*‐test was performed in (C) and (G). Ratio paired *T*‐test was performed in (F).

Liver fibrosis has long been believed to be an irreversible pathological problem that determines the long‐term outcome and mortality of chronic inflammatory liver diseases. However, recent therapeutic advances in chronic liver disease have suggested that fibrosis and fibrolysis are dynamic processes that move in two directions [6]. The origin of cells triggering fibrosis signaling in chronic liver injury, including viral, alcoholic, and autoimmune hepatitis, is still unclear, although the primary target cell of the above injury would be hepatocytes, which often promote the fibrogenic process resulting from complex cell‐cell interactions initiated within hepatocytes. Importantly, a positive correlation has been reported between p16^Ink4a^+ hepatocytes and the degree of liver fibrosis in liver samples from NAFLD patients [11, 14]. We first established a mouse model of liver fibrosis model to investigate the role of p16^Ink4a^+ hepatocytes in the initiation of liver fibrogenic processes. Chronic liver injury was induced by repeated injections of CCl_4_, which subsequently led to the development of liver fibrosis (Figure 1A) [15]. Based on serum AST and ALT test results, a single dose of CCl_4_ induced severe acute liver injury (Figure S1A). However, after four weeks of injections, the degree of liver injury caused by each dose of CCl_4_ became relatively mild, with the injury gradually resolving between days 3 and 5 after injection (Figure S1A). In both conditions, the expression of p16^Ink4a^ in hepatocytes increased after CCl_4_ injections. However, in the chronic liver injury with 4‐week CCl_4_ treatment, p16^Ink4a^ expression did not recover to normal levels (Figure S1B,C), indicating the persistent presence of p16^Ink4a^+ hepatocytes.

We conducted p16‐tdTomato (p16‐Tom) mice to label the cells with p16^Ink4a^ expression by tamoxifen (TAM) administration. As the duration of CCl_4_ exposure increased, the number of tdTomato‐positive (Tom+) hepatocytes (Figure S1D) and the degree of fibrosis progressively increased, showing a positive correlation (Figure 1B,C). Because of the concomitant expansion of Tom+ hepatocytes and liver fibrosis (Figure 1D; Figure S1E), to further confirm their sustained expression of p16^Ink4a^, we collected Tom+ and Tom‐ hepatocytes 10 days after the final administration of CCl_4_ (Figure 1E). Higher levels of p16^Ink4a^, Tom, and Cre^ERT2^ expressions were observed in the Tom+ hepatocytes (Figure 1F), which were referred to as p16^h^ hepatocytes in this study.

To further investigate the transcriptomic characteristics of p16^h^ hepatocytes, we performed RNA‐seq analysis on Tom+ and Tom‐ hepatocytes from both the oil and CCl_4_ groups. Principle component analysis results revealed that CCl_4_ treatment significantly altered gene expression in Tom+ and Tom‐ hepatocytes (Figure S2A,B). However, when we analyzed the differentially expressed genes (DEGs), we found that zonation markers (e.g., Glul, Lgr5, Cyp2e1, Hal, and Sds) [16] were significantly involved (Figure S2C). Spatial analysis of Tom+ cells revealed that they were predominantly localized to zone 3 (Figure 1G), the region most affected by CCl_4_‐induced injury. This spatially asymmetric distribution prevented bulk RNA‐seq from distinguishing the transcriptomic characteristics of p16^h^ hepatocytes from the influence of zonation.

### 2.2 Transcriptomic Features of p16^Ink4a^+ Hepatocytes

**FIGURE 2:**
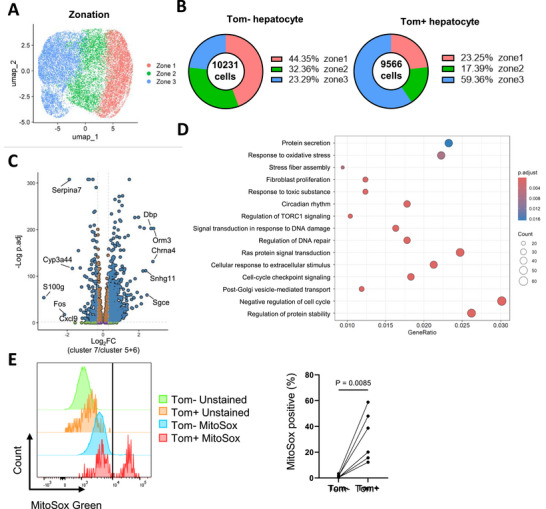
The scRNA‐seq analysis reveals the transcriptomic features of p16^h^ mouse hepatocytes in liver fibrosis model (A) UMAP showing the zonation of hepatocytes isolated from the mice treated with CCl_4_ for 4 weeks, following the schedule shown as in Figure 1A. (B) The population of Tom+ and Tom‐ hepatocytes in each zone shown in (A). (C) Volcano plot showing the differentially expressed genes (DEGs) between cluster 7 and clusters 5 and 6 within zone 3. DEGs were identified by log_2_FC > 0.3 or < ‐0.3 and *p*‐values < 0.05 adjusted by B–H method. (D) Gene ontology (GO) analysis revealing the enriched biological process terms in the up‐regulated DEGs of cluster 7 compared with clusters 5 and 6. All terms were identified by *p*‐values < 0.05 adjusted by B–H method. (E) MitoSOX staining results of hepatocytes isolated from mice treated as in Figure 1A analyzed by FACS. Representative histograms and the threshold were shown in the left panel. The amounts of MitoSOX‐positive hepatocytes were quantified in the right panel (n=5). Ratio paired *T*‐test was performed in (E).

Zonation is a continuous spectrum, and it is difficult to isolate cells from specific zones without the use of transgenic mice [17]. Therefore, we used fixed single‐cell RNA sequencing (scRNA‐seq) to selectively analyze p16^h^ hepatocytes, including the cells from different zonation recognized by the marker gene expressions of zone 1 (Sds and Cyp2f2), zone 2 (Hamp and Igfbp2), and zone 3 (Glul and Cyp2e1) (Figure 2A; Figure S3A–C). The expression level of Tom could be correctly reflected in Tom+ and Tom‐ hepatocytes (Figure S3D). Consistent with the results from tissue sections, the increased p16^h^ hepatocytes in the liver fibrosis model were predominantly located in zone 3 (Figure 2B). Interestingly, unsupervised clustering revealed that many p16^h^ cells were relatively enriched in cluster 7 compared with clusters 5 and 6, suggesting that, despite being hepatocytes from zone 3, a subset of p16^h^ hepatocytes exhibited unique transcriptional characteristics (Figure S3E).

Compared to other hepatocytes in zone 3, cluster 7 hepatocytes showed significant upregulation of 2169 genes and downregulation of 230 genes (Figure 2C). GO analysis revealed that these genes were associated with phospholipid and steroid metabolism (Figure S4A). Consistent with this, impaired lipid homeostasis is closely associated with liver fibrosis [18]. In addition to metabolism‐related terms, cell cycle arrest, oxidative stress response, DNA damage repair, and protein secretion were also enriched in upregulated genes of cluster 7 (Figure 2D). Among them, we identified genes that were specifically upregulated in zone 3 Tom+ hepatocytes (Figure S4B). These genes were further confirmed by qPCR (Figure S4C) and have been reported to be involved in fibrosis [19, 20, 21], exosome formation [22, 23], cell cycle arrest [24, 25], and oxidative stress [26, 27].

Similarly, gene set variation analysis (GSVA) results similarly indicated that cluster 7 exhibited signatures of inflammation, protein secretion, and reduced E2F targets (Figure S5). Furthermore, mitochondrial ROS levels were significantly higher in the Tom+ hepatocytes (Figure 2E), which was also described in hepatocytes from aged mice [28, 29]. These findings suggested that the CCl_4_‐induced p16^h^ hepatocytes may represent oxidative stress‐induced senescent cells [30] and facilitate liver fibrosis.

### 2.3 Specific Elimination of p16^h^ Hepatocytes Ameliorates Liver Fibrosis

**FIGURE 3:**
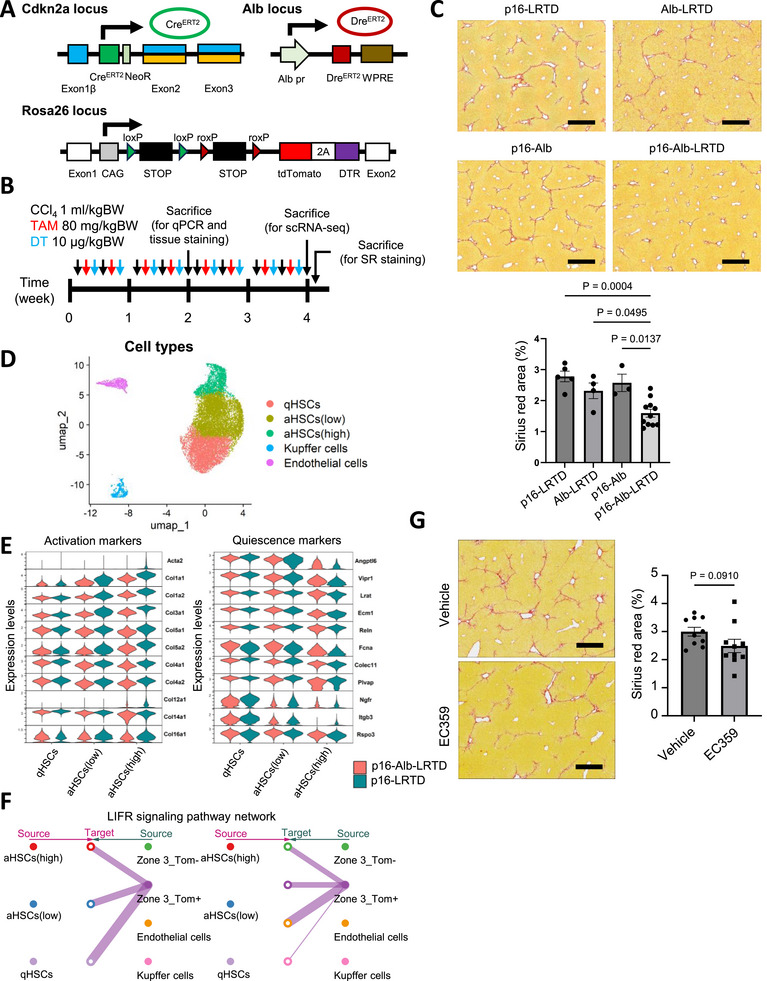
Specifically eliminating p16^h^ hepatocytes improves CCl_4_‐induced liver fibrosis (A) The design construction of p16‐Alb‐LRTD transgenic mouse model. (B) The schematic image of CCl_4_, TAM, and diphtheria toxin (DT) administration for eliminating p16^h^ hepatocytes. (C) Sirius red staining results of the liver following 4‐week treatment, as shown in (B). The representative images were shown in the upper panels. Scale bar, 500 µm. Quantitative results of the Sirius red area percentage were shown in the lower panel. (p16‐LRTD n=5, Alb‐LRTD n=4, p16‐Alb n=3, p16‐Alb‐LRTD n=11) (D) UMAP showing cell types and activation status of hepatic satellite cells (HSCs) isolated from p16‐LRTD and p16‐Alb‐LRTD mice treated as shown in (B). (E) Violin plots showing the expression levels of activation and quiescence marker genes in HSCs. (F) Connection plot showing the LIFR signaling network between HSC and hepatocyte datasets shown in Figure 2A. (G) Sirius red staining results of the liver following 4‐week treatment of CCl_4_ with vehicle or 5 mg/kgBW of EC359. The representative images were shown in the left panel. Scale bar, 500 µm. (n=10) One‐way ANOVA followed by Tukey's test (C) and unpaired *t*‐test (G) were performed.

It has been widely accepted that cellular senescence is involved in the progression of liver fibrosis [31]. However, recent studies have increasingly shown that senescent cells of different cell types appear to have different functions within this highly dynamic process and may even have opposing effects on disease progression or aging [13, 32, 33, 34]. Therefore, to uncover the unique role of p16^h^ hepatocytes in the initiation of liver fibrogenic processes, we generated a p16‐Cre^ERT2^/Alb‐Dre^ERT2^/R26‐CAG‐LSL‐RSR‐tdTomato‐2A‐DTR (p16‐Alb‐LRTD) mouse model to selectively label and eliminate p16^h^ hepatocytes after TAM and diphtheria toxin (DT) administration (Figure 3A,B) [35]. Immunofluorescence staining validated the cell type‐specific labeling and clearance efficiency of p16^h^ hepatocytes (Figure S6A,B). We found a significant suppression of liver fibrosis in a p16‐Alb‐LRTD group compared to the littermate control after identical treatments (Figure 3C). Although the expression of Acta2 in hepatic stellate cells (HSCs) did not change markedly, the levels of typical collagens and fibrosis were also significantly decreased following the 2‐week treatment (Figure S6C,D) [36].

We performed scRNA‐seq on HSCs from the above two groups of mice and categorized the HSCs into three subgroups based on activation status (Figure 3D; Figure S7A–C). In the activated HSCs (aHSCs), the p16‐Alb‐LRTD group exhibited significantly lower levels of activation‐associated genes and higher expression of quiescent marker genes compared to the littermate controls (Figure 3E) [37]. This indicates that the removal of p16^h^ hepatocytes effectively reduced overall collagen production by HSCs. These findings are consistent with the tissue Sirius red staining results.

We integrated data from Tom+ and Tom‐ hepatocytes in zone 3 with data from HSCs to explore a total of 43 potential ligand‐receptor interactions between cell populations [38]. The signaling pathways in which p16^h^ hepatocytes act as ligand‐secreting cells and HSCs serve as the receptor‐expressing counterparts were focused. Surprisingly, the only pathway that showed a difference between Tom+ and Tom‐ hepatocytes was the leukemia inhibitory factor receptor (LIFR) pathway, which was delivered from Tom+ hepatocytes to HSCs (Figure 3F). The LIFR pathway belongs to an IL‐6 family, and the receptor (Lifr and Il6st (gp130)) or ligand (cardiotrophin 1 (Ctf1) and cardiotrophin‐like cytokine factor 1 (Clcf1)) expression was confirmed in HSCs and hepatocytes, respectively (Figure S7D,E) [39]. LIF family has been reported to promote fibrosis in the vascular system, kidney, and hepatocellular carcinoma [40, 41, 42], and we found mRNA levels of Lif and Ctf1 were up‐regulated in Tom+ hepatocytes (Figure S7F). Treatment of LIFR inhibitor, EC359, also showed a trend toward reduced fibrosis, but not statistically significant in the CCl_4_‐treated mice (Figure 3G).

To clarify whether senescent hepatocytes up‐regulated fibrogenic phenotypes of HSC through LIF or CTF1, we conducted Huh7 cells as an in vitro platform [43, 44, 45]. We first confirmed that Huh7 cells enter a senescent state following doxorubicin treatment, which was evaluated by SA‐β‐gal staining and p21 expression (Figure S8A,B). We further observed a significant up‐regulation of secreted cytokines such as LIF, CTF1, and IL6. Treatment with either a p38 inhibitor or a JAK inhibitor significantly suppressed the expression of these genes, indicating that both p38 activation and autocrine feedback contributed to the up‐regulation of IL‐6 family cytokines (Figure S8C).

To further confirm the biochemical activity conservation of LIF and CTF1 across human and mouse, we treated primary mouse HSCs and human HSC line LX‐2 with recombinant mouse CTF1, human CTF1, or human LIF. We found that all three cytokines induced STAT3 phosphorylation in both cell types (Figure S8D), indicating that the signaling activity of these cytokines was conserved between humans and mice. Besides, condition media from senescent Huh7 activated collagen expression in both mHSCs and LX‐2 cells, which was alleviated by JAK inhibitor and LIFR inhibitor, EC359, indicating that the LIFR‐JAK pathway contributed to the cross‐talk between senescent hepatocytes and HSCs (Figure S8E,F).

### 2.4 Hepatocytes in Cirrhosis Patients Show p16^h^ Characteristics

**FIGURE 4:**
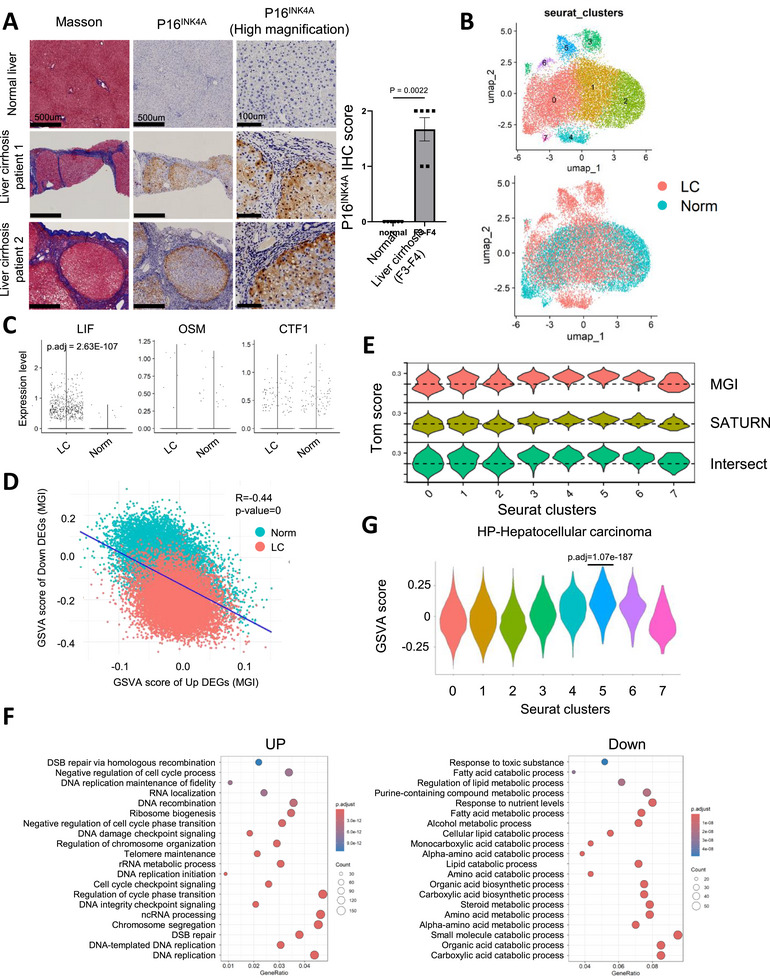
Hepatocytes expressing p16^INK4A^ positively correlate with liver cirrhosis in patients (A) Masson's trichrome and IHC staining of p16 in the livers from patients. The liver samples were obtained from resected tissues of patients with colorectal cancer liver metastases or liver cirrhosis classified as normal or severe fibrosis (F3 and F4), respectively (n=6). Representative images were shown in the left panels. Scale bar, 500 µm, and 100 µm for high magnification. Scoring of p16 IHC was shown in the right panel. 0: negative, 1: weak positive, 2: strong positive. (B) UMAP showing clusters and patients of hepatocytes isolated from normal and liver cirrhosis (LC) biopsies. (C) Violin plots showing the expression levels of indicated genes. (D) Scatter plots showing the GSVA scores of up‐regulated and down‐regulated DEG lists derived from the comparison within mouse cluster 7 and cluster 5+6 in human hepatocytes scRNA‐seq datasets. The mouse DEG lists were converted by the MGI method to human gene names. Pearson correlation coefficient was calculated and shown on the figure. (E) Violin plots showing Tom score representing the difference of GSVA scores converted by the indicated methods. (F) Gene ontology (GO) analysis revealing the enriched biological process terms in the up‐ or down‐regulated DEGs of cluster 5 compared with other cells. DEGs were identified by log_2_FC > 0.3 or < ‐0.3 and *p*‐values < 0.05 adjusted by B–H method. All terms were identified by *p*‐values < 0.05 adjusted by B–H method. (G) Violin plots showing GSVA scores of the indicated gene set from MSigDB. Mann–Whitney test was performed in (A).

To verify whether p16^h^ hepatocytes are also associated with fibrogenic promotion in humans, we first analyzed the relationship between p16 expression levels and fibrosis severity in human cirrhosis samples. Compared to normal liver tissue, samples from cirrhotic patients showed a significantly higher abundance of p16‐expressing hepatocytes (Figure 4A; Figure S9A). We then performed scRNA‐seq analysis on hepatocytes and showed that only a subset of cells from cirrhotic patients overlapped with normal hepatocytes and that clusters 3–6 represented cirrhosis‐specific populations independent of zonation (Figure 4B; Figure S9B,C). Although we cannot draw a clear conclusion about p16^Ink4a^ expression because CDKN2A does not exclusively encode p16^Ink4a^, it was significantly increased in hepatocytes from cirrhotic samples (Figure S9D) consistent with the results of immunohistochemical analysis (Figure 4A). Related to the LIFR pathway, human hepatocytes from liver cirrhosis patients showed higher levels of another LIFR family ligand, LIF, but not CTF1, compared with hepatocytes from normal patients (Figure 4C).

To identify cells in the human transcriptome with high similarity to mouse p16^h^ hepatocytes, we employed two approaches based on (1) gene sequences (MGI database) [46] or (2) protein sequence similarity followed by deep learning (SATURN algorithm) [47]. Using these methods, we converted the up and down‐regulated genes from mouse cluster 7 within zone 3 into their human homologous gene names (Figure S10A). The GSVA scores of the up and down gene lists in each approach showed a significant negative correlation (Figure 4D; Figure S10B). By calculating the differences in GSVA scores, we defined a “Tom score” and found that, regardless of the conversion approach, clusters 3–6 showed a higher similarity to mouse p16^h^ hepatocytes (Figure 4E). When cells were classified into high, medium, and low Tom score groups, cluster 5 was identified as the population with the largest number of mouse p16^h^‐like hepatocytes (Figure S10C,D). GO analysis revealed that this cluster had lost the original metabolic functions of hepatocytes and exhibited several transcriptional profiles resembling those of premalignant cells, which was consistently confirmed by the GSVA result of hepatocellular carcinoma‐related gene set (Figure 4F,G) [48].

To determine whether the association between p16^h^ hepatocytes and pre‐malignant lesions can be reproduced in a model of chronic liver injury, we treated p16‐Tom mice with CCl_4_ injections for up to 24 weeks. Around the 12th week, p16^h^ hepatocytes were labeled via TAM administration (Figure S11A). At this point, p16^h^ hepatocytes were predominantly observed as single, scattered cells throughout the liver (Figure S11B). However, following an additional 12 weeks of CCl_4_ injections, we found that two tumor lesions harbor the clone that originated from p16^h^ hepatocytes (Figure S11C). This indicates that the p16^h^ hepatocytes present at the 12th week have potential for malignant transformation. Therefore, p16^h^ hepatocytes in both the murine liver fibrosis model and human cirrhotic samples showed consistency in promoting fibrosis, possibly through the LIFR signaling pathway. The transcriptomic comparison also suggests a potential association between p16^h^ hepatocytes and premalignant lesions.

## 3 Discussion

In this study, we found the accumulation of p16^h^ hepatocytes in the long‐term CCl_4_‐induced liver injury. In contrast to our findings, a recent study using similar p16^Ink4a^ tracer mice reported that p16‐positive hepatocytes were barely detectable in CCl_4_‐induced liver injury [13]. This difference may be determined by the threshold of p16^Ink4a^ promoter activity in the tracer mice used. In this regard, it should be noted that in both tracer mouse models Neo cassette gene, which acts as a local enhancer to augment promoter activity without compromising tissue p16^Ink4a^ promoter fidelity [49, 50, 51], was inserted at the different sites of the p16^Ink4a^ locus. When the Neo cassette gene was inserted between downstream of the p16^Ink4a^ promoter and upstream of the reporter gene, the expression of the reporter gene was much more enhanced, while accurately correlating with the expression of endogenous p16^Ink4a^ [49]. The level of enhancement appeared to be dependent on the insertion site at the p16^Ink4a^ locus (our mouse model inserted the Neo gene at exon 1a, and the mouse model reported by Zhao et al. inserted the Neo gene at exon 3 of the Cdkn2a locus). This idea is strongly supported by observations that mouse Tom+ hepatocytes in CCl_4_‐induced liver injury expressed significantly higher levels of p16^Ink4a^ mRNA and that much higher numbers of p16^Ink4a^+ hepatocytes were detected in human liver samples from cirrhotic patients [11, 14].

The functional zonation of hepatocytes is now a well‐established concept, whose differences are determined by several factors, such as oxygen and nutrient levels throughout the liver lobule [52, 53]. Hepatocytes in zone 1 carry out most of the liver's metabolic functions, such as lipid metabolism, urea and protein synthesis, and gluconeogenesis, whereas those in zone 3 are characterized by glycolysis, glutamine synthesis, and xenobiotic biotransformation reactions. These findings further support our observation that the accumulation of p16^h^ hepatocytes was predominantly detected in zone 3 of CCl_4_‐induced liver injury because CCl_4_ is a classic zone 3 hepatotoxin that causes liver injury leading to hepatic failure.

Single‐cell transcriptomics revealed that p16^h^ hepatocytes were enriched in a unique cluster in liver injury that shared several characteristics of senescence and aging, including upregulation of genes involved in cell cycle arrest, oxidative stress response, DNA damage repair, and protein secretion. GSVA analysis also revealed that this cluster shared signatures of inflammation, protein secretion, and reduced E2F targets. Importantly, mitochondrial ROS levels were significantly higher in the Tom+ hepatocytes. ​A similar set of transcriptomic features has been identified in hepatocytes from aged mice [28], suggesting that aged hepatocytes may possess an enhanced capacity to promote fibrosis upon liver injury. Taken together, CCl_4_‐induced p16^h^ hepatocytes may represent oxidative stress‐induced senescent cells. This is further supported by the fact that CCl_4_ is often used to induce oxidative stress [54].

By introducing the dual Cre and Dre model [35], we designed the p16‐Alb‐LRTD mice to specifically eliminate p16^h^ hepatocytes. In this model, p16^h^ hepatocytes consistently accumulated in zone 3 following the CCl_4_ treatment as observed in the p16‐Tom model. Furthermore, we found that this model effectively improved CCl_4_‐induced fibrosis in the region surrounding central veins (zone 3). Meanwhile, we observed that a 4‐week treatment led to very mild fibrosis in zone 1 and zone 2 of only Alb‐LRTD and p16‐Alb‐LRTD mice. We speculate that this phenomenon may be attributed to the incomplete suppression of DTR leakage expression by loxP‐Stop‐loxP. This intrinsic limitation of the dual model restricts its applicability for investigating more long‐term liver fibrosis. Single‐cell transcriptomic analysis of HSCs after ablation of p16^h^ hepatocytes in CCl_4_‐induced liver injury clearly demonstrated the suppression of many HSCs activation‐related gene expression. These results suggest that p16^h^ hepatocytes act as an epistasis for HSCs activation, which promotes liver fibrosis. Then, we identified Ctf1, a LIFR pathway member, as a crosstalk signaling between p16^h^ hepatocytes and HSCs. Consistent with this, LIFR has recently been proposed to amplify pathogenic activation of fibroblasts and induce various organ fibrosis [41]. Indeed, LIFR inhibition suppressed the collagen expression in primary HSCs promoted by secreted factors from senescent Huh7 cells. However, LIFR expressed by hepatocytes was previously mentioned, which is involved in cross‐talk between hepatocytes and neutrophils and accelerates liver regeneration [55]. Therefore, LIFR inhibition itself may show the less‐than‐expected improvement in the chronic CCl_4_ injury model.

A similar accumulation of p16^h^ hepatocytes was also observed in liver samples from cirrhotic patients. We also identified LIF, another member of the LIFR pathway, as a potential activating factor for human HSCs and was up‐regulated in senescent Huh7 with p38‐ and/or JAK‐dependent mechanisms. Surprisingly, the MGI database and deep learning‐based algorithm, SATURN, identified a hepatocyte cluster from cirrhosis patients that was most similar to the mouse unique cluster containing a large number of p16^h^ cells. This cluster exhibited several premalignant‐like transcriptomic signatures, including the highest GSVA score of hepatocellular carcinoma gene set, suggesting that p16^h^ hepatocytes have the potential to transform into liver cancer cells under specific conditions. However, it is important to note that the clonal expansion observed in mice remains an observational finding at this stage, and further studies will be required to elucidate the molecular or pathological mechanism. A very recent study has also demonstrated that senescent hepatocytes in a metabolic‐associated steatohepatitis (MASH) state partially serve as an origin for hepatocellular carcinoma by downregulating p53 and FBP1 [56].

In conclusion, we uncovered the molecular crosstalk between hepatocytes and HSCs in the CCl_4_‐induced liver injury model, which ultimately induces liver fibrosis. Although CCl_4_‐induced liver injury model does not well reflect the pathological status of liver cirrhosis patients, when integrating transcriptomic data from CCl_4_‐treated livers with those from cirrhotic patients, we were still able to project murine p16^h^ hepatocytes onto human cirrhosis‐specific clusters. In this scenario, p16^h^ hepatocytes play an important role in activating HSCs and promoting fibrogenic processes through LIFR signaling, suggesting that these pathways may be a promising therapeutic target (Figure S11D). In addition, p16^h^ hepatocytes may provide a clue to understanding the molecular basis underlying the association between liver fibrosis and hepatocellular carcinogenesis.

### 3.1 Resource Availability

The scRNA‐seq datasets and bulk RNA‐seq datasets described in this article have been uploaded to the Gene Expression Omnibus (GEO) with accession number GSE312804, GSE312805, GSE312806, and GSE312919. All other data needed to evaluate the conclusions of the study are present in the article and/or supplementary figures. Source data is available from the corresponding authors upon reasonable request.

## 4 Materials and Methods

### 4.1 Mouse Model

All mice were C57BL/6 background and housed in a temperature‐controlled (23–25°C) and humidity‐controlled colony room, maintained on a 12‐h light/dark cycle (lights on from 08:00 to 20:00). They were provided with standard food (CA‐1, CLEA Japan) and water ad libitum. All animals were handled according to the Guidelines for Animal Experiments of the Institute of Medical Science, the University of Tokyo, and Institutional Laboratory Animal Care. All animal experiments were approved by the Animal Experiment Committee at IMSUT (A16‐33, A21‐26). All p16‐Tom male mice used for labeling p16^h^ cells were heterozygous and generated by crossing p16‐Cre^ERT2^ and R26‐CAG‐LSL‐tdTomato‐WPRE mice described previously [57]. All p16‐Alb‐LRTD male mice used for specifically eliminating p16^h^ hepatocytes were also heterozygous by crossing p16‐Cre^ERT2^, Alb‐Dre^ERT2^ (Shanghai Model Organisms Center, Inc., NM‐KI‐200076), and R26‐CAG‐LSL‐RSR‐tdTomato‐2A‐DTR (Shanghai Model Organisms Center, Inc., NM‐KI‐190086). For all the mice we used as a result of in‐house mating.

To induce liver fibrosis, CCl_4_ (KANTO CHEMICAL) was diluted to 20% (v/v) with corn oil (Wako) and administered to mice via intraperitoneal injection every three days at a dose equivalent to 1 mL/kgBW of CCl_4_. Tamoxifen (Cayman) and diphtheria toxin (Wako) were administered at 80 mg/kgBW and 10 µg/kgBW, respectively, to stabilize Cre^ERT2^ and Dre^ERT2^ or eliminate the DTR‐expressing cells [35]. EC359 was dissolved in 10% DMSO+ 40% PEG300+ 5% Tween‐80+ 45% saline and intraperitoneally injected into mice with 5 mg/kgBW one day prior to each CCl_4_ injection.

### 4.2 Primary Mouse Hepatocyte Isolation

The method of hepatocyte isolation was previously described [58]. Mice were anesthetized using 2,2,2‐tribromoethanol. The portal vein was transected after inserting a 22G indwelling needle into the inferior vena cava. The EGTA solution (HBSS(‐) (Wako) + 0.5 mm EGTA + 10 mm HEPES (Nacalai Tesque)) and collagenase solution (HBSS(+) (Wako) + 1 mg/mL collagen Type IV (Gibco), 10 mm HEPES) were perfused sequentially at a flow rate of 5 mL/min for 5 and 7 min, respectively. The liver tissues were gently dissociated in PBS and filtered through a 70 µm cell strainer, followed by pelleting hepatocytes with three centrifugations at 50 × g for 2 min.

For the MitoSOX staining, isolated hepatocytes were stained with 5 µm MitoSOX Green (Invitrogen, M36006) at 37°C for 30 min, and then analyzed by BD FACSAria III (BD Biosciences).

### 4.3 Primary Mouse Hepatic Stellate Cells (HSCs) Isolation

The method and solution compositions for hepatic stellate cells were previously described [59]. Mice were anesthetized, and the portal vein was transected after inserting a 22G indwelling needle into the inferior vena cava. The EGTA, pronase, and collagenase solutions were perfused sequentially at a flow rate of 5 mL/min for 3, 5, and 7 min, respectively. The liver tissue was collected and stirred at 37°C in pronase/collagenase solution for 25 min, maintaining a pH of 7.4. The digested tissue was filtered through a 100 µm cell strainer. After washing with GBSS/B solution (Sigma), the hepatic stellate cells were isolated by density gradient separation using Nycodenz (Serumwerk) solution.

### 4.4 Histology of Mouse Samples

Anesthesia was conducted, followed by systemic perfusion with PBS (Nacalai tesque). For the preparation of paraffin blocks, livers were fixed in 10% formalin for 24 h. After formalin fixation, dehydration was performed using 70% ethanol, and paraffin blocks were subsequently prepared. Sections with a thickness of 5 µm were cut from the paraffin blocks.

For the Sirius red staining, paraffin‐embedded sections were deparaffinized and stained at room temperature using Picro‐Sirius Red Solution (abcam) for 1 h. The sections were then washed three times with 0.5% acetic acid solution and dehydrated. The sections were imaged using a BZ‐X810 (Keyence) with a 20×/0.75 objective lens. Tiled images were captured in a 5 × 5 grid, and the area of Sirius Red staining was quantified by using the BZ‐800Z analyzer software (ver 1.1.2.4).

For the immunohistostaining, paraffin‐embedded sections were deparaffinized and subjected to antigen retrieval using either enzymatic treatment with 10 µg/mL proteinase K (37°C, 10 min) or autoclaving in Sodium Citrate Antigen Retrieval Buffer (Proteintech; 120°C, 20 min). Subsequently, blocking was performed at room temperature with 10% normal goat serum (Nichirei Biosciences). The sections were incubated overnight at 4°C with primary antibodies, including anti‐mCherry (abcam, ab205402; 1:250 dilution), anti‐CYP2E1 (abcam, ab28146; 1:250 dilution), and anti‐HNF4α (CST, 3113S; 1:250 dilution). Secondary antibodies were applied at room temperature for 1 h, including anti‐chicken AF488 (abcam, ab150169; 1:500 dilution) and anti‐rabbit AF647 (Invitrogen, A21245; 1:500 dilution). Finally, nuclei were counterstained with DAPI (CST), and the sections were imaged using a BZ‐X810 (Keyence). For the immunohistostaining with DAB, after antigen retrieval, the sections were incubated with 0.3% H_2_O_2_ for 30 min to block endogenous peroxidase activity. Primary antibody against RFP (Rockland 600‐401‐379, 1:250) was applied and incubated overnight at 4°C. After washing, N‐Histofine Simple Stain Mouse MAX‐PO (R) (Nichirei Biosciences, 414341) was used and incubated for 30 min at room temperature. Signal was developed using Histofine DAB substrate (Nichirei Biosciences, 415171) for 10 min. Hematoxylin counterstaining was performed by incubating the sections for 1 min, followed by dehydration and mounting.

For frozen sections, livers were fixed in 4% paraformaldehyde in PBS (Nacalai Tesque) for 24 h and then cryoprotected in a 30% sucrose solution. After sucrose substitution, the tissue was embedded in an Optimal Cutting Temperature Compound (Sakura Finetek), and sections with a thickness of 10 µm were prepared. The nuclei were stained with Hoechst 33342 (Thermo Fisher), and the imaging was conducted using a BZ‐X810 (Keyence). The tdTomato‐positive hepatocytes were quantified from at least 5 randomly selected images using the BZ‐800Z analyzer.

### 4.5 Histology of Human Liver Biopsy

The use of human liver tissue was approved by the Ethics Committee of the Tokyo Metropolitan Cancer and Infectious Diseases Center Komagome Hospital (approval numbers: 3101, 3200). Liver specimens were obtained from resected tissues (non‐tumorous regions) of patients with colorectal cancer liver metastases or liver cysts (F0) and patients with liver fibrosis (F3 or F4) and hepatocellular carcinoma accompanied by liver cirrhosis (F4, one sample among them was used for scRNA‐seq). Following pathological examination, the specimens were classified as normal liver tissue and severely fibrotic tissue (F3 or F4), respectively. The Pathology Department of the Tokyo Metropolitan Cancer and Infectious Diseases Center Komagome Hospital performed tissue sectioning, immunostaining, and evaluation entirely. Immunostaining was performed using the BOND‐III system (Leica Biosystems), with antigen retrieval using BOND Epitope Retrieval Solution 1 (Leica Biosystems). The p16 immunostaining was applied by conducting CINtec p16 Histology (Roche, 825‐4713; 1:2.5 dilution). The single‐blind evaluation was based on the p16 staining score, assigning a score of 0 for negative, 1 for weak positive, and 2 for strong positive for each specimen.

For the Masson‐Noguchi staining, tissue sections were deparaffinized and stained with a mixed solution of equal volumes of 10% Trichloroacetic (KANTO CHEMICAL) acid and 10% Potassium dichromate (KANTO CHEMICAL) for 10 min, followed by Weigert's iron hematoxylin (MUTO PURE CHEMICALS) for 10 min. Afterward, the sections were treated with 1% hydrochloric acid alcohol, rinsed, and washed in 1% acetic acid water, and incubated in a mixed solution of ponceau xylidine (0.6 g, Sigma), acid fuchsin (0.2 g, MERCK), Acid Red 1 (0.1 g, Tokyo Chemical Industry), and acetic acid (1 mL) dissolved in 500 mL of distilled water for 20 min. This was followed by a wash in 1% acetic acid water and treatment with 2.5% phosphotungstic acid (KANTO CHEMICAL) for 5 min. The sections were stained with 0.04% aniline blue (Nacalai Tesque) for 5 min, washed in 1% acetic acid water, dehydrated through graded alcohols, cleared in xylene, and mounted.

### 4.6 Measurement of AST and ALT Levels in Serum

Blood was taken from the mice through the inferior vena cava and left at room temperature for 1 h. To obtain serum, samples were centrifuged at 2000 g for 20 min. Serum levels of AST and ALT were measured by using the SPOTCHEM EZ SP‐4430 (Arkray) analyzer with SPOTCHEM II (Arkray).

### 4.7 Cell Culture

Huh7 and LX‐2 cells were cultured in DMEM low glucose and high glucose (nacalai tesque), respectively, with 10 % fetal bovine serum (FBS) (Nichirei) and 1X antibiotic‐antimycotic (nacalai tesque) at 37°C under 5 % CO_2_ with normoxia. Primary mouse HSCs were cultured in collagen I‐coated plates (Corning) and DMEM high glucose with 10 % FBS and 1X antibiotic‐antimycotic at 37°C under 5 % CO_2_ with hypoxia conditions (5% O_2_). Senescent Huh7 cells were induced by treating the cells with 0.2 µm doxorubicin for two days, followed by 0.1 µm BI2536 for another two days, and then culturing them in standard medium for an additional two days [43, 44, 45]. Senescence was assessed using the Senescence β‐Galactosidase Staining Kit (Cell Signaling Technology) according to the manufacturer's protocol. Stained cells were imaged and quantified using the BZ‐X810 microscope (Keyence). CYT387 (Selleck), SB203580 (Selleck), and EC359 (MedChemExpress) were used at a concentration of 2 µm, 10 µm, and 100 nm, respectively. Recombinant human LIF (R&D), human CTF1 (Peprotech), and mouse CTF1 (Peprotech) were treated at 25, 100, and 100 ng/ml, respectively.

### 4.8 RNA Isolation and Quantitative Real‐Time PCR

Isolated cells and tissues obtained from mice were lysed in TRIzol (Invitrogen), followed by mixing with chloroform (FUJIFILM) at a ratio of 5:1 (TRIzol: chloroform). After centrifugation at 12 000 g for 15 min at 4°C, the aqueous phase was collected and mixed with an equal volume of 70% ethanol. RNA was further purified by loading to columns from the RNeasy Mini Kit (QIAGEN) or the Single Cell RNA Purification Kit (Norgen). Reverse transcription was performed with ReverTra Ace qPCR RT Master Mix (TOYOBO) or SuperScript IV VILO Master Mix (Thermofisher) to synthesize complementary DNA (cDNA) for qPCR. Real‐time qPCR was conducted using THUNDERBIRD SYBR Next qPCR Master Mix (TOYOBO) on the StepOnePlus system (Applied Biosystems).

### 4.9 Analysis of Bulk RNA‐Sequencing

The Tom+ and Tom‐ hepatocytes were isolated from male p16‐Tom mice treated with vehicle (corn oil) or CCl_4_ using BD FACSAria III (BD Biosciences). Dead cells were removed by DAPI staining (CST). Total RNA was extracted using the Single Cell RNA Purification Kit (Norgen). Library preparation, sequencing, and quality checks were conducted by Novogene Inc. In brief, mRNA was enriched by polyA, strand‐specific RNA‐seq libraries were prepared, and sequencing was performed on the NovaSeq 6000 system (Illumina). Reads were quality‐checked (fastp v0.23.2), aligned to the mm10 genome (HISAT2 v2.1.0) [60], and TPM values were calculated with TPMcalculator (v0.0.4) [61]. Principal component analysis (PCA) was performed using R (v4.3.2), with genes showing TPM expression levels of 1 or higher in at least 6 samples. Differentially expressed genes (DEGs) were identified using DESeq2 (v1.42.1), and the Volcano Plot was generated with GraphPad Prism (v9.4.1).

### 4.10 Single‐Cell RNA‐Sequencing (scRNA‐seq) Library Preparation

To prepare hepatocyte suspensions of CCl_4_‐treated male p16‐Tom mice, PBS was perfused, followed by liver excision and chopping into 5 mm^2^ pieces. The use of human liver samples was approved by the Ethics Committee of the Tokyo Metropolitan Cancer and Infectious Diseases Center Komagome Hospital (approval number: 3200). Human liver samples were processed within 2 h of surgical resection by sectioning a 2 cm × 2 cm tissue fragment into 5 mm^2^ pieces. The tissue fragments were fixed in Fix & Perm Buffer (10X Genomics) containing 4% formaldehyde (Polysciences) at 4°C with moderate agitation for 16–20 h. Fixation was stopped by adding 1.25 m glycine, and the fragments were washed with tissue resuspension buffer (0.496X PBS (Nacalai Tesque), 50 mm Tris‐HCl pH 8.0, 0.02% (w/v) BSA (Sigma), and RNase inhibitors (TOYOBO). The tissues were dissociated into single cells using a gentleMACS Dissociator (Miltenyi) with 0.2 mg/mL of Liberase TL and TH (Roche). Dissociated cells were washed with tissue resuspension buffer, filtered through a 70 µm cell strainer, and stained with MitoBrilliant 646 (Tocris Bioscience, 1:5000 dilution) at 37°C for 30 min. Following staining, the cells were washed, and filtered, and nuclei were stained with 2 µg/mL of Hoechst dye (Dojindo).

The cell suspension was analyzed using a BD FACSAria III flow cytometer (BD Biosciences), and Hoechst+ /MitoBrilliant 646+ /Tom+ or Tom‐ hepatocytes were collected in Protein LoBind Tubes (Eppendorf). Library preparation for single‐cell transcriptomes of isolated hepatocytes was performed using the Chromium Fixed RNA Kit (10X Genomics, Mouse or Human Transcriptome) following the manufacturer's protocol. Briefly, fixed RNA fragments within the cells were captured via hybridization with Chromium Mouse/Human Transcriptome Probe Set v1.0.1 (10X Genomics), and single‐cell droplets were generated using the Chromium iX (10X Genomics). In the mouse samples, three pairs of customized tdTomato probes were added to the probe set for quantifying tdTomato expression. Probe sequences: tdTomato‐1 TACAGGAACAGGTGGTGGCGGCCCT, TTTACGGATGTCAACGTCACAC, tdTomato‐2 AACACAGTGCACACCACGCCACGTT, GCCTGACAACGGGCCACAACTCCTC, tdTomato‐3 AGGACGTCCCGCGCAGAATCCAGGT, GGCAACACAGGCGAGCAGCCAAGGA.

Sequencing of the libraries was performed using DNBSEQ‐G400RS (MGI Tech) with 150‐bp paired‐end reads. MGI FASTQ data were converted to Illumina‐compatible FASTQ format and demultiplexed by sample barcodes using SplitDualBarcodes.pl (provided by MGI). Gene expression matrices were generated using the cellranger multi of CellRanger (v7.2.0 for mouse and v7.0.1 for human) with the mm10 or h38 reference genome.

For the hepatic stellate cells isolation from p16‐Alb‐LRTD and p16‐LRTD mice, the library preparation followed the manufacturer's protocol for the Single Cell 3’ Reagent Kit v3.1 (10x Genomics). Quality control and sequencing of the samples were performed by Novogene Inc. (China). Library sequencing was conducted using the NovaSeq X Plus system (Illumina) with 150‐bp paired‐end reads. Gene expression matrices were generated using the cellranger count of CellRanger (v7.0.1) with the mm10 reference genome.

### 4.11 Analysis of Mouse scRNA‐seq Datasets

Data processing, quality control, normalization, visualization, and clustering were basically performed using Seurat package (v5.1.0) [62] in R (v.4.3.2). For the mouse hepatocytes datasets, cells with a mitochondrial gene fraction exceeding 0.05, fewer than 2000 genes, greater than 10 000 genes, or UMI counts greater than 100 000 were excluded due to low‐quality cells or doublets. Following normalization, scaling, dimensional reduction, and clustering, clusters with low gene counts or expressing Ptprc, Dcn, or Pecam1 were further excluded to focus exclusively on hepatocytes. Differentially expressed genes (DEGs) analysis was conducted using the FindMarkers function with Log2FC>0.3 and FDR<0.05 adjusted by B–H method. Gene ontology (GO) analysis of DEGs and gene set variation analysis (GSVA) were performed using clusterProfiler (v4.10.1) [63] and GSVA (v1.47.3) [64] packages, respectively. The gene sets for GSVA were obtained from MSigDB v2023.1.mm, and all GSVA score comparisons within clusters were conducted using *t*‐tests to calculate *p*‐values, subsequently adjusted by the B–H method for multiple testing correction.

For the mouse HSC datasets, cells with a mitochondrial gene fraction exceeding 0.05, fewer than 2000 genes, greater than 7000 genes, or UMI counts greater than 50 000 were excluded due to the low‐quality cells or doublets. The cells obtained from p16‐LRTD and p16‐Alb‐LRTD mice were integrated by using the reciprocal PCA method with 30 dimensions. Clusters with low gene counts were further excluded. The expression levels of quiescent and activating marker genes were considered to determine the activity of HSCs [37]. Cell‐cell interaction analysis using hepatocyte and HSC datasets was performed using the CellChat package (v1.6.1) [38].

### 4.12 Analysis of Human scRNA‐seq Datasets

For the human hepatocyte datasets, cells with a mitochondrial gene fraction exceeding 0.05, fewer than 1500 genes, greater than 6000 genes, or UMI counts greater than 40 000 were excluded due to the low‐quality cells or doublets. The cells obtained from normal livers and cirrhotic livers were integrated by using the reciprocal PCA method with 30 dimensions. Clusters with low gene counts or expressing PTPRC, DCN, or PECAM1 were excluded to focus exclusively on hepatocytes. DEGs analysis was conducted using the FindMarkers function with Log2FC>0.3 and FDR<0.05 adjusted by B–H method.

The up‐regulated and down‐regulated DEG lists were generated by comparing cluster 7 with clusters 5 and 6 of mouse hepatocyte datasets and further converted to human gene names in two methods to identify human hepatocytes sharing transcriptomic similarities with p16^h^ mouse hepatocytes. In the first method, according to the vertebrate homology dataset obtained from MGI, which is derived from Alliance of Genome Resources [46], the mouse gene symbols were converted to human gene symbols based on the consistency of DB.Class.Key.

In the second method, we applied the SATURN (Species Alignment Through Unification of RNA and Proteins) algorithm to construct a macrogene framework for integrating human and mouse genes [47]. Macrogenes are defined as groups of genes that share similar protein embeddings derived from large protein models, which predict protein structures from amino acid sequences. Gene expression profiles are transformed into macrogene expressions through gene‐to‐macrogene weights, which are learnable parameters of the SATURN algorithm. In addition, SATURN constructs a joint cell embedding space (macrogene embedding), in which the same cell types are aligned across species by encoding macrogene expressions into a lower‐dimensional space. SATURN employs a two‐step training process consisting of pretraining and fine‐tuning. In the pretraining step, the gene‐to‐macrogene weights are optimized to reconstruct gene expression profiles. In the fine‐tuning step, the macrogene embeddings are optimized to discriminate cell types across species, while the gene‐to‐macrogene weights are kept frozen. For the application of SATURN in converting between human and mouse genes through macrogene weights, the pretraining step can be omitted.

Similar gene pairs between human and mice were identified by evaluating the cosine similarity of their weight vectors derived from the pretrained gene‐to‐macrogene weights. The weight vector for a given gene *i* was represented as $\vec{W_{i}}={\left(W_{1,i},W_{2,i},\text{…},W_{M,i}\right)}^{T}$, where *W*_*m*,*g*_ represents the gene‐to‐macrogene weights (*m*  =  1, …, *M*,  *g*  =  1, …, 2*G*). The cosine similarity between a mouse gene *i* and a human gene *j* is calculated as follows:

(1) $$similarity=\frac{{\vec{W}}_{i}^{\left(mouse\right)}\cdot {\vec{W}}_{j}^{\left(human\right)}}{\left\|{\vec{W}}_{i}^{\left(mouse\right)}\right\|\left\|{\vec{W}}_{j}^{\left(human\right)}\right\|}$$

Following the SATURN methodology, macrogene weights are initialized using the ESM2 model (a protein embedding model). During pretraining, SATURN employs an autoencoder optimized with a zero‐inflated negative binomial (ZINB) loss, denoted as $L_{\mathrm{rc}}$​, to reproduce gene expression profiles. Additionally, a supplementary loss term, Ls, is introduced to ensure that the macrogene weights capture similarities in the protein embedding space. For pretraining, a subset of 12 000 highly variable human and mouse genes (G) was utilized, and the number of macrogenes (M) was 5000. The pretraining procedure consisted of 2000 steps. For the 12 000 highly variable human and mouse gene pairs, a pair of genes was considered similar if their cosine similarity exceeded 0.85. This filtering step ensures that only gene pairs with high similarity in the embedding space were retained for further analysis, facilitating robust comparisons between human and mouse genes.

The converted up‐regulated and down‐regulated gene lists were used to calculate the GSVA scores in the human hepatocyte datasets, and the difference values of the two scores were defined as Tom scores to represent the similarity to p16^h^ mouse hepatocytes.

### 4.13 Protein Extraction and Immunoblotting

Collected hepatocytes were lysed by using Laemmli buffer (2% SDS, 10% glycerol, 5% 2‐mercaptoethanol, 0.002% bromophenol blue, and 62.5 mm Tris‐HCl at pH 6.8). Cell lysates were separated by SDS‐PAGE and transferred to PVDF membranes (Millipore) and then subjected to immunoblotting with the anti‐p16 (abcam, ab211542, 1:1000 dilution), anti‐STAT3 (CST, 12640, 1:1000 dilution), and anti‐p‐STAT3 (CST, 9145, 1:1000 dilution) using the ECL detection system. Anti‐GAPDH (CST, 5174S, 1:1000 dilution) or anti‐ACTB (Santa Cruz, sc‐69879, 1:5000 dilution) was used as a loading internal control. Membranes were supplied with Immobilon Forte Western HRP substrate (Millipore) and detected using Amersham Imager 680 (Cytiva).

### 4.14 Statistical Analysis

For animal experiments, all the male mice were randomly assigned to each group and independently followed the same schedule in each experimental design. The sample sizes were not predetermined by pilot studies. The single‐blind design was only employed to qualify for p16 staining in the patient pathological sections. Data are presented as means ± SEM unless otherwise noted. Comparisons between the two groups were made by paired or unpaired two‐tailed Student's *t*‐test. Multi‐comparisons of one‐variable data were carried out by one‐way analysis of variance (ANOVA) followed by a Sidak test or Dunnett's test. All analyses were performed using GraphPad Prism 9 (v9.4.1) or R.

## Author Contributions

M.N. and Y.J. conceived the project. K.N., Y.J., T.‐W.W., and M.N. planned the experiments. K.N., T.‐W.W., S.K., and S.T. performed the experiments. K.N., T.‐W.W., S.K., S.T., Y.T.O., K.Y., T.K., M.Kimura, T.H., S.O., T.I., T.C., T.J., M.Katano, K.Kamataki, R.Y., E.S., K.Kimura, S.Y., S.I., Y.F., A.M., Y.J., and M.N. analyzed the results. M.N., K.N., and T.‐W.W. wrote the paper, with editing by all the other authors.

## Conflicts of Interest

M.N. is the scientific advisor and shareholder at reverSASP Therapeutics. S.Y. is co‐founder of Celaid Therapeutics. T.I., M.Katano, and K.Kamataki are employees of GMO Internet Group, Inc. T.C. and T.J. are employee and CEO of GMO Research Activity Support & Technology, Inc., respectively. R.Y. is an employee of GMO Healthtech, Inc.

## Supporting information




**Supporting File 1**: advs73862‐sup‐0001‐SuppMat.docx.

**Supporting File 2**: advs73862‐sup‐0002‐FiguresS1.pptx.

**Supporting File 3**: advs73862‐sup‐0003‐TableS2.docx.

## Data Availability

The data that support the findings of this study are available from the corresponding author upon reasonable request.
