# Supplementary material for: p16Ink4a‐Positive Hepatocytes Drive Liver Fibrosis Through Activation of LIFR Family Pathway
Source: Adv Sci (Weinh). 2026 Jan 25;13(17):e10562. doi: 10.1002/advs.202510562 (PMC13042423; doi:10.1002/advs.202510562)
Supplement: Supplementary file 1 — Supporting File 1: advs73862‐sup‐0001‐SuppMat.docx. [file ADVS-13-e10562-s003.docx]

**Figure S1. Chronic liver injury leads to the prolonged expression of p16^Ink4a^ in hepatocytes**

(A) The serum levels of AST and ALT, (B) mRNA levels of p16^Ink4a^, and (C) protein levels of p16^INK4A^ at designated days after the final CCl_4_ treatment. The CCl_4_ injection schedule for the 4-week treatment was shown in Figure 1A. The corn oil group was sacrificed one day after the final dose of injection. (D) Immunofluorescent staining of indicated antibodies in the liver samples treated as shown in Figure 1A. Scale bar: 100 μm. (E) Gating strategy of FACS analysis shown in Figure 1D. The Tom fluorescent images of sorted Tom+ and Tom- hepatocytes were shown in the lower-right panel. Scale bar: 100 μm. One-way ANOVA followed by Dunnett’s test was performed in (A) and (B).

**Figure S2. A bulk transcriptome analysis reveals that Tom+ hepatocytes tend to localize within zone 3**

(A) The PCA plot illustrating the transcriptomic distribution of hepatocytes. Tom+ and Tom- hepatocytes were isolated from livers treated with corn oil or CCl_4_ as shown in Figure 1A. (B and C) Volcano plots showing the DEGs between Tom+ and Tom- hepatocytes from (B) corn oil and (C) CCl_4_ treated mice. DEGs were identified by log_2_FC > 0.75 or < -0.75 and p-values < 0.05 adjusted by B-H method.

**Figure S3. The zone 3 localization of Tom+ hepatocytes is recapitulated by scRNA-seq analysis**

(A) The gating strategy of FACS for sorting fixed single Tom+ and Tom- hepatocytes isolated from the mice treated as shown in Fig. 1A. (B) Feature plots showing the expression levels of indicated zonation marker genes in the scRNA-seq datasets. (C) UMAP showing the distribution of Tom+ and Tom- hepatocytes. (D) Violin plot showing the expression level of Tom detected by customized probes in this study. (E) UMAP showing the Seurat clustering of hepatocytes in the left panel. The percentage of Tom+ cells within clusters in zone 3 were calculated and shown in the right panel.

**Figure S4. DEG analysis of Tom+ hepatocytes in zone 3**

(A) Gene ontology (GO) analysis revealing the enriched biological process terms in the up- or down-regulated DEGs of cluster 7 compared with clusters 5+6. DEGs were identified by log_2_FC > 0.3 or < -0.3 and p-values < 0.05 adjusted by B-H method. All terms were identified by p-values < 0.05 adjusted by B-H method. (B) Violin plots showing the expression levels of indicated up-regulated DEGs in cluster 7 across all zones. (C) qPCR analysis of indicated genes in isolated Tom+ and Tom- hepatocytes. (n=5) Ratio paired T-test was performed.

**Figure S5. GSVA analysis of Tom+ hepatocytes in zone 3**

GSVA results of indicated terms from hallmark or Reactome databases. Violin plots displayed the distribution of GSVA score of each zone or cluster in zone 3. Feature plots revealed the scores of each cell, and the black arrowhead indicated the cluster 7.

**Figure S6. Elimination of p16^h^ hepatocytes reduced collagen expression in livers**

(A) Frozen sections of livers from mice with indicated genotypes and treatments shown in Figure 3B. Scale bars, 500 μm in the upper panel and 100 μm in the lower panel. White arrowheads indicated Tom+ hepatocytes. CV: central vein. (B) The quantitative results of sections shown in (A). (n=3) (C) qPCR analysis of indicated genes in whole liver lysates from the mice with indicated genotypes following the treatments shown in Figure 3B. (n=5) (D) Sirius red staining results of the liver following 2-week treatment as shown in Figure 3B. The representative images were shown in the left panels. Scale bar, 500 μm. Quantitative results of Sirius red area percentage were shown in the right panel. (n=5) One-way ANOVA followed by the Sidak test was performed in (B), and an unpaired T-test was performed in (C) and (D).

**Figure S7. The scRNA-seq analysis of HSCs reveals the improvement of CCl_4_-induced liver fibrosis**

(A) UMAP showing the distribution of single-cell transcriptome from indicated mice. (B and C) Feature plots showing the expression levels of (B) cell type marker genes, (C) HSC activation markers, and quiescence markers. (D) Violin plots showing the expression levels of receptors in LIFR pathway of indicated cell types in mouse HSC datasets. (E) Violin plots showing the expression levels of ligands in LIFR pathway of indicated zonation in mouse hepatocyte datasets. (F) qPCR analysis of indicated genes in isolated Tom+ and Tom- hepatocytes. (n=5) Ratio paired T-test was performed.

**Figure S8. LIFR-JAK pathway is involved in mediating the profibrotic effects of senescent hepatocytes on HSCs**

(A) SA-β-gal staining results of proliferating (Prol) and senescent (Sene) Huh7 induced by treating 0.2 μM doxorubicin. The representative images were shown in the left panel. Scale bar, 50 μm.(B) qPCR analysis of p21 in Huh7 cells under indicated conditions. (n=3) (C) qPCR analysis of indicated genes in Prol and Sene Huh7 cells with 48 hours treatments of indicated inhibitors. (n=3) (D) Immunoblotting results of mouse CTF1 (100 ng/ml), human CTF1 (100 ng/ml) and human LIF (25 ng/ml) treated HSCs using indicated antibodies. Cell lysates were collected 30 or 60 minutes after treatments. (E and F) qPCR analysis of indicated genes in (E) mouse HSCs and (F) LX-2 cells treated with conditional media (C.M.) collected from Prol and Sene Huh7 cells for 24 hours. Inhibitors were added to the conditional media with indicated concentration. (n=3) Unpaired T-test (A and B), and one-way ANOVA followed by the Tukey’s test (C, E, and F) were performed.

**Figure S9. Hepatocytes expressing p16^INK4A^ are enriched in liver cirrhosis patients**

(A) Representative images of scoring p16 IHC staining shown in Figure 4A. (B) The gating strategy of FACS for sorting fixed single human hepatocytes. (C) Feature plots showing the expression levels of indicated genes. (D) Violin plot showing the expression levels of CDKN2A.

**Figure S10. Human hepatocytes similar to mouse p16^h^ hepatocytes are identified through distinct gene conversion approaches**

(A) The Venn diagrams illustrating the number of genes identified and the overlap between the two methods of gene name conversion. (B) Scatter plots showing the GSVA scores of up-regulated and down-regulated DEG lists derived from the comparison within mouse cluster 7 and cluster 5+6 in human hepatocytes scRNA-seq datasets. The mouse DEG lists were converted by SATURN or the intersect between MGI and SATURN to human gene names. Pearson correlation coefficient was calculated and shown on the figure. (C) Histograms showing the distribution of Tom scores in the human hepatocyte datasets. The threshold for distinguishing between low and medium levels was determined by the intersection point of the two peaks, while the threshold for separating medium and high levels was defined as the 90th percentile of the distribution in Norm group. (D) Barplot showing the proportion of cells with high, medium, and low Tom scores in the indicated clusters.

**Figure S11. Mouse p16^h^ hepatocytes potentially associates with premalignant lesions**

(A) The schematic image of CCl_4_ and TAM administration. (B) Representative images of H&E, Sirius Red, and anti-RFP immunohistochemistry staining of p16-Tom mice in 12w group as shown in (A). Scale bar: 1 mm (C) Representative images of photo, H&E, Sirius Red, and anti-RFP immunohistochemistry staining of p16-Tom mice in 24w group as shown in (A). Tumor lesions were indicated with black arrowheads. In the images with high magnification, the boundary between tumor and normal area was assigned with dash line. Scale bars: 5 mm (photo), 1 mm (low magnification), and 200 μm (high magnification). (D) Schematic figure of brief summary in this study.
