# Supplementary material for: p16Ink4a‐Positive Hepatocytes Drive Liver Fibrosis Through Activation of LIFR Family Pathway
Source: Adv Sci (Weinh). 2026 Jan 25;13(17):e10562. doi: 10.1002/advs.202510562 (PMC13042423; doi:10.1002/advs.202510562)
Supplement: Supplementary file 2 — Supporting File 2: advs73862‐sup‐0002‐FiguresS1.pptx. [file ADVS-13-e10562-s002.pptx]

## Slide 1
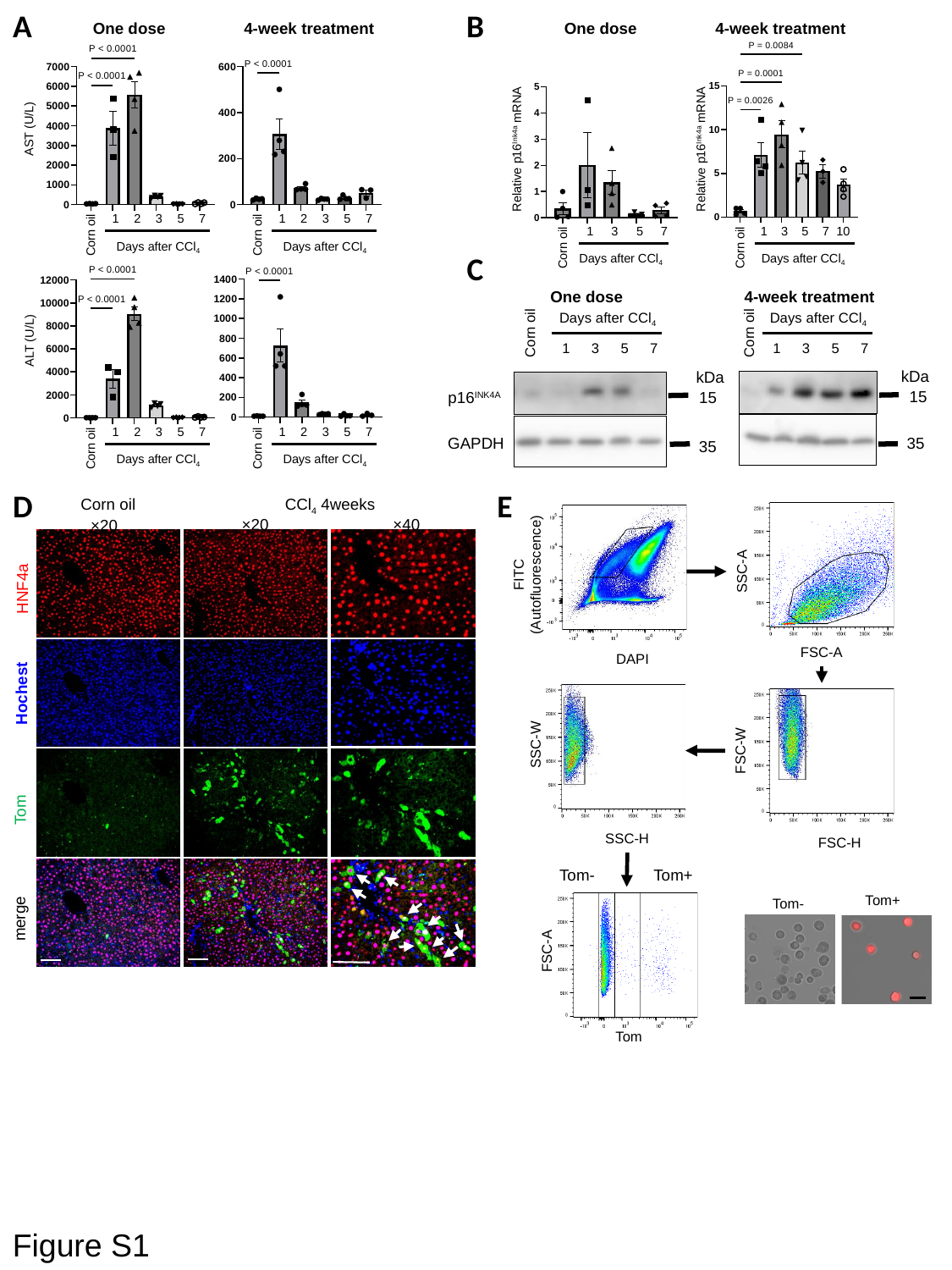

A
B
One dose
4-week treatment
One dose
4-week treatment
Relative p16Ink4a mRNA
Relative p16Ink4a mRNA
1
3
5
7
1
3
5
7
10
Corn oil
Corn oil
Days after CCl4
Days after CCl4
AST (U/L)
1
2
3
5
7
1
2
3
5
7
Corn oil
Corn oil
Days after CCl4
Days after CCl4
C
1
2
3
5
7
1
2
3
5
7
Corn oil
Corn oil
Days after CCl4
Days after CCl4
ALT (U/L)
One dose
4-week treatment
Days after CCl4
Days after CCl4
Corn oil
Corn oil
1
3
5
7
1
3
5
7
kDa
kDa
15
p16INK4A
15
35
GAPDH
35
D
E
FITC
(Autofluorescence)
SSC-A
FSC-A
DAPI
SSC-W
FSC-W
SSC-H
FSC-H
Tom-
Tom+
Tom+
Tom-
FSC-A
Tom
Corn oil
CCl4 4weeks
×20
×40
×20
HNF4a
Hochest
Tom
merge
Figure S1

## Slide 2
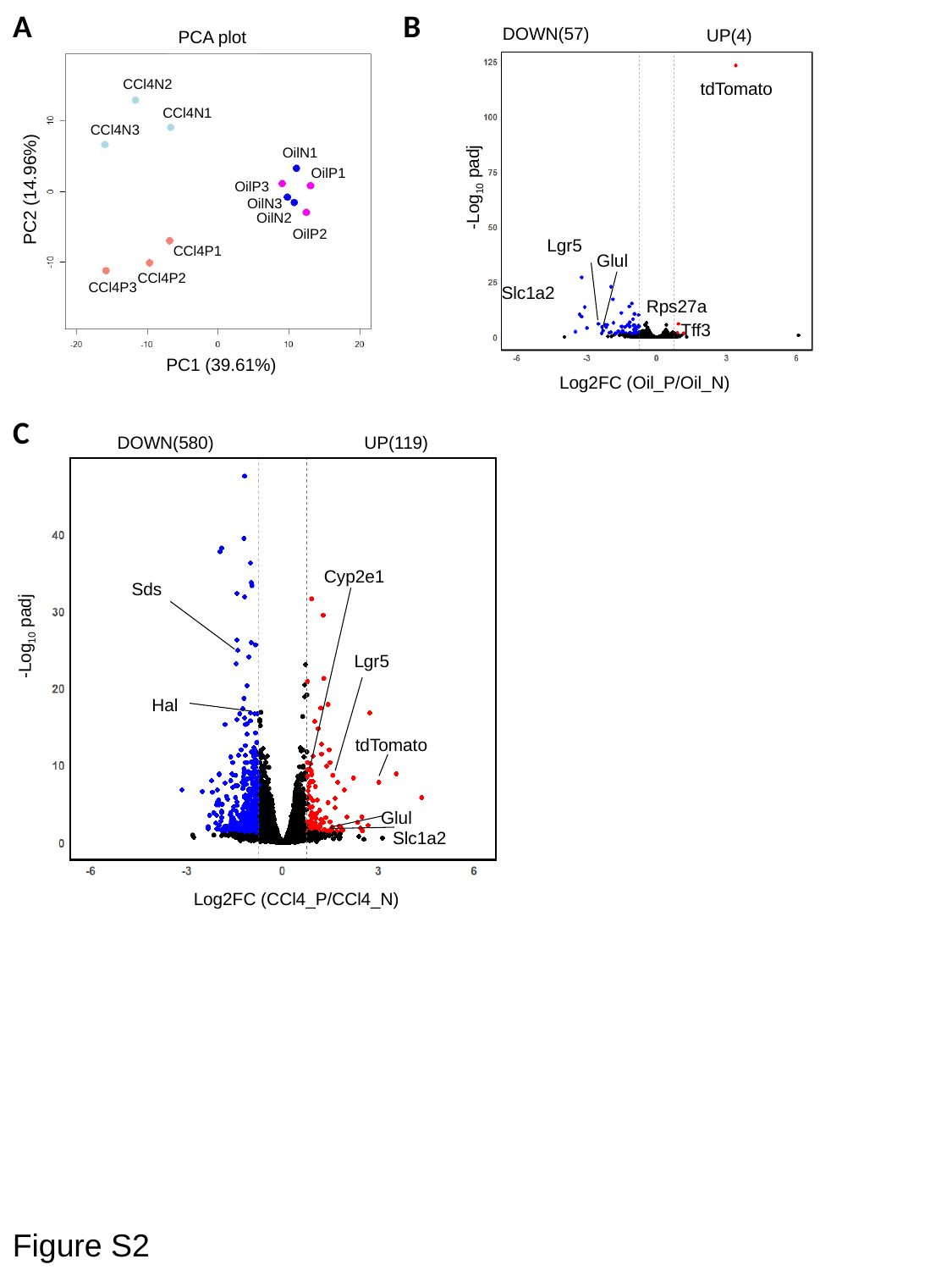

A
B
CCl4N2
CCl4N1
CCl4N3
OilN1
OilP1
OilP3
OilN3
OilN2
OilP2
CCl4P1
CCl4P2
CCl4P3
PCA plot
PC2 (14.96%)
PC1 (39.61%)
DOWN(57)
UP(4)
tdTomato
-Log10 padj
Lgr5
Glul
Slc1a2
Rps27a
Tff3
Log2FC (Oil_P/Oil_N)
C
DOWN(580)
UP(119)
Cyp2e1
Sds
-Log10 padj
Lgr5
Hal
tdTomato
Glul
Slc1a2
Log2FC (CCl4_P/CCl4_N)
Figure S2

## Slide 3
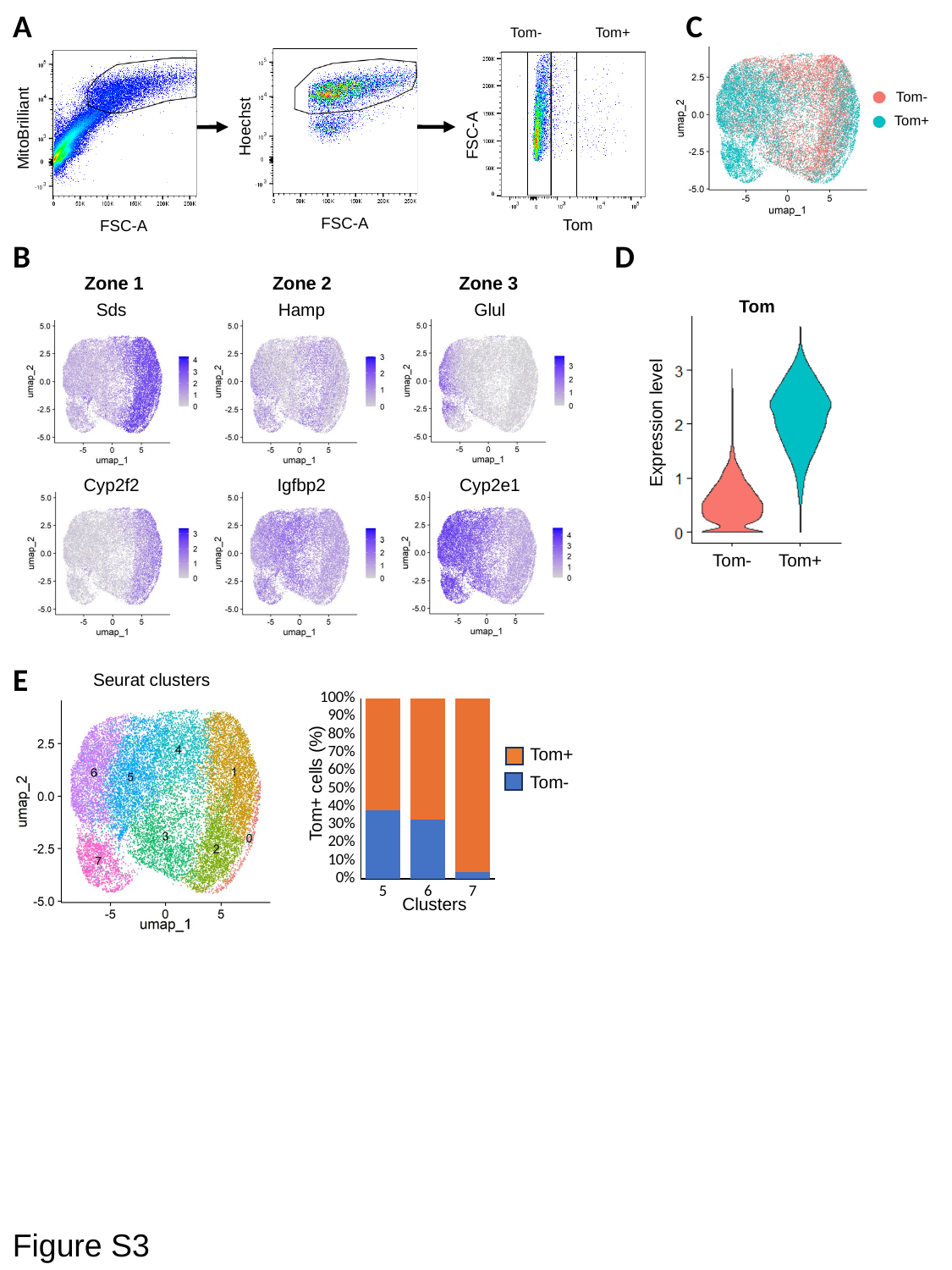

A
C
Tom-
Tom+
Hoechst
FSC-A
MitoBrilliant
FSC-A
Tom
FSC-A
Tom-
Tom+
B
D
Zone 1
Zone 2
Zone 3
Tom
Sds
Hamp
Glul
Expression level
Cyp2f2
Igfbp2
Cyp2e1
Tom-
Tom+
E
Seurat clusters
### Chart
| Category | Tom- | Tom+ |
|---|---|---|
| 5 | 1237.0 | 2006.0 |
| 6 | 1092.0 | 2227.0 |
| 7 | 54.0 | 1445.0 |
Tom+
Tom-
Tom+ cells (%)
Clusters
Figure S3

## Slide 4
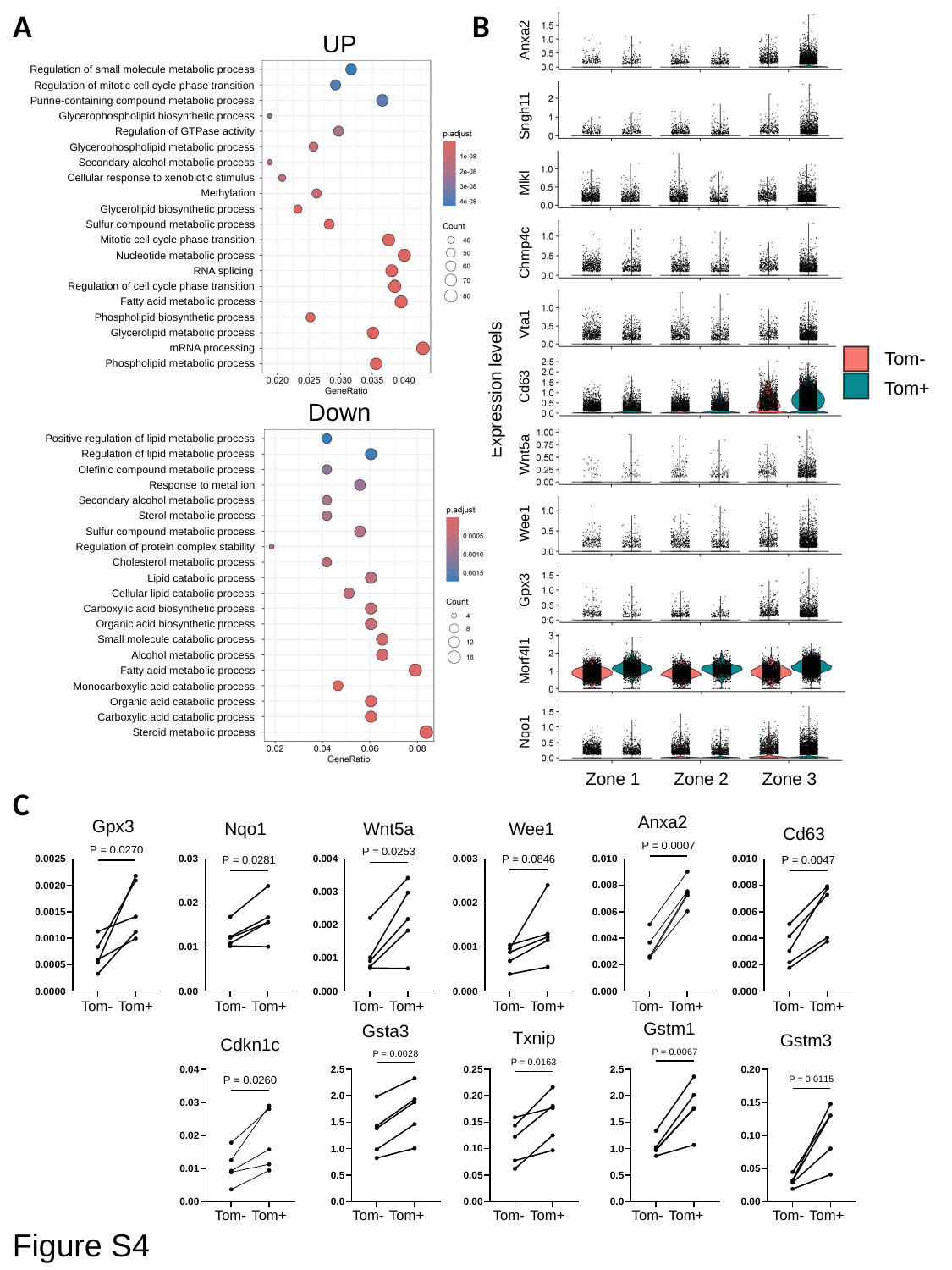

A
B
Anxa2
Sngh11
Mlkl
Chmp4c
Vta1
Cd63
Expression levels
Wnt5a
Wee1
Gpx3
Morf4l1
Nqo1
Zone 1
Zone 2
Zone 3
UP
Regulation of small molecule metabolic process
Regulation of mitotic cell cycle phase transition
Purine-containing compound metabolic process
Glycerophospholipid biosynthetic process
Regulation of GTPase activity
Glycerophospholipid metabolic process
Secondary alcohol metabolic process
Cellular response to xenobiotic stimulus
Methylation
Glycerolipid biosynthetic process
Sulfur compound metabolic process
Mitotic cell cycle phase transition
Nucleotide metabolic process
RNA splicing
Regulation of cell cycle phase transition
Fatty acid metabolic process
Phospholipid biosynthetic process
Glycerolipid metabolic process
mRNA processing
Phospholipid metabolic process
Down
Positive regulation of lipid metabolic process
Regulation of lipid metabolic process
Olefinic compound metabolic process
Response to metal ion
Secondary alcohol metabolic process
Sterol metabolic process
Sulfur compound metabolic process
Regulation of protein complex stability
Cholesterol metabolic process
Lipid catabolic process
Cellular lipid catabolic process
Carboxylic acid biosynthetic process
Organic acid biosynthetic process
Small molecule catabolic process
Alcohol metabolic process
Fatty acid metabolic process
Monocarboxylic acid catabolic process
Organic acid catabolic process
Carboxylic acid catabolic process
Steroid metabolic process
Tom-
Tom+
C
Anxa2
Gpx3
Nqo1
Wnt5a
Wee1
Cd63
Tom-
Tom-
Tom-
Tom-
Tom-
Tom-
Tom+
Tom+
Tom+
Tom+
Tom+
Tom+
Gstm1
Gsta3
Txnip
Gstm3
Cdkn1c
Tom-
Tom-
Tom-
Tom-
Tom-
Tom+
Tom+
Tom+
Tom+
Tom+
Figure S4

## Slide 5
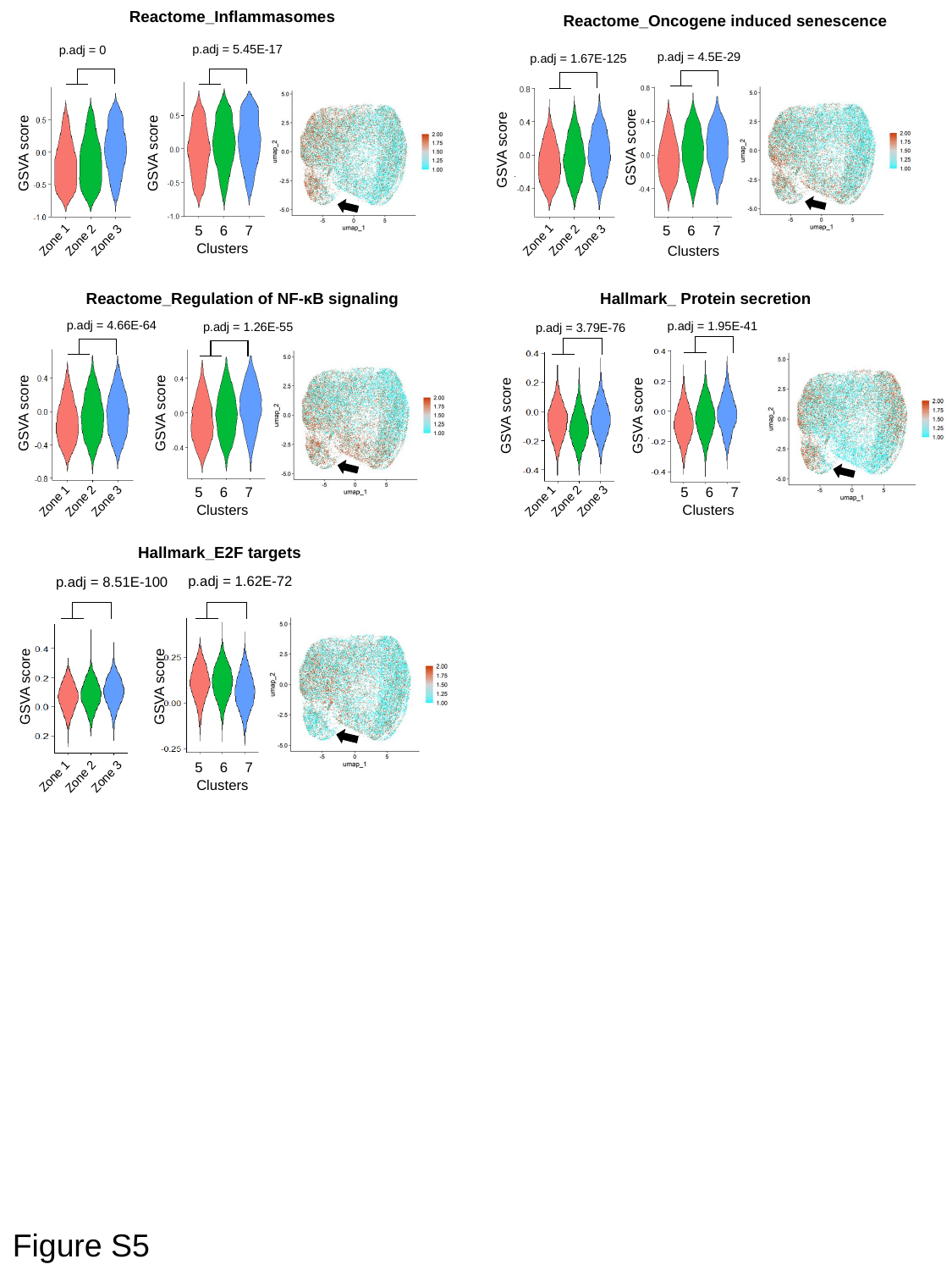

Reactome_Inflammasomes
p.adj = 5.45E-17
p.adj = 0
GSVA score
GSVA score
5
6
7
Zone 1
Zone 3
Zone 2
Clusters
Reactome_Oncogene induced senescence
p.adj = 4.5E-29
GSVA score
GSVA score
5
6
7
Zone 3
Zone 2
Clusters
p.adj = 1.67E-125
Zone 1
Reactome_Regulation of NF-κB signaling
p.adj = 4.66E-64
p.adj = 1.26E-55
GSVA score
GSVA score
5
6
7
Zone 1
Zone 3
Zone 2
Clusters
Hallmark_ Protein secretion
p.adj = 1.95E-41
p.adj = 3.79E-76
GSVA score
GSVA score
5
6
7
Zone 1
Zone 3
Zone 2
Clusters
Hallmark_E2F targets
p.adj = 1.62E-72
p.adj = 8.51E-100
GSVA score
GSVA score
5
6
7
Zone 1
Zone 3
Zone 2
Clusters
Figure S5

## Slide 6
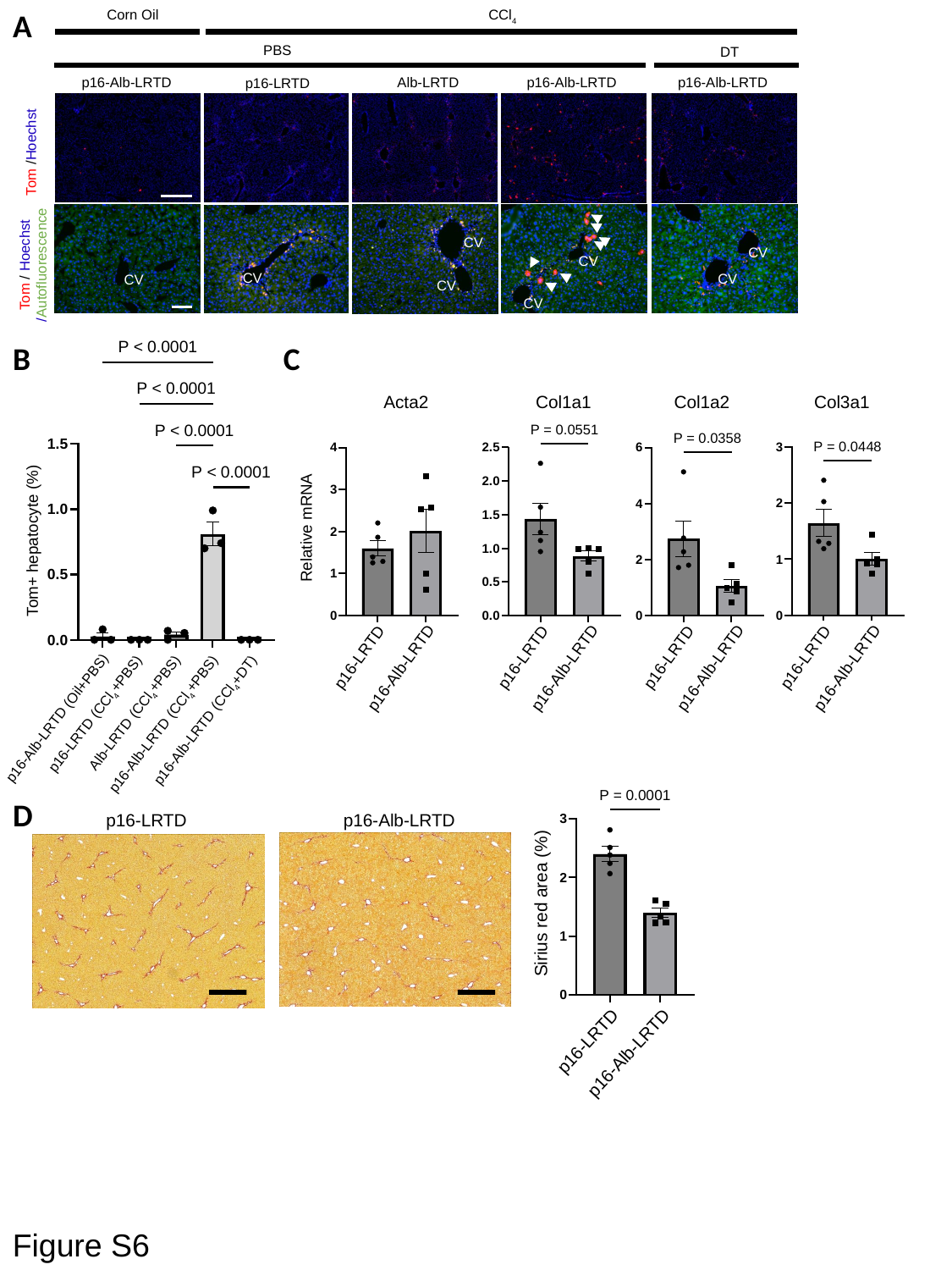

A
Corn Oil
CCl4
PBS
DT
p16-Alb-LRTD
Alb-LRTD
p16-Alb-LRTD
p16-Alb-LRTD
p16-LRTD
Tom /Hoechst
CV
CV
Tom / Hoechst /Autofluorescence
CV
CV
CV
CV
CV
CV
Tom+ hepatocyte (%)
p16-Alb-LRTD (Oil+PBS)
p16-LRTD (CCl4+PBS)
Alb-LRTD (CCl4+PBS)
p16-Alb-LRTD (CCl4+PBS)
p16-Alb-LRTD (CCl4+DT)
B
C
Acta2
Col1a1
Col1a2
Col3a1
Relative mRNA
p16-LRTD
p16-LRTD
p16-LRTD
p16-LRTD
p16-Alb-LRTD
p16-Alb-LRTD
p16-Alb-LRTD
p16-Alb-LRTD
p16-Alb-LRTD
p16-LRTD
Sirius red area (%)
p16-LRTD
p16-Alb-LRTD
D
Figure S6

## Slide 7
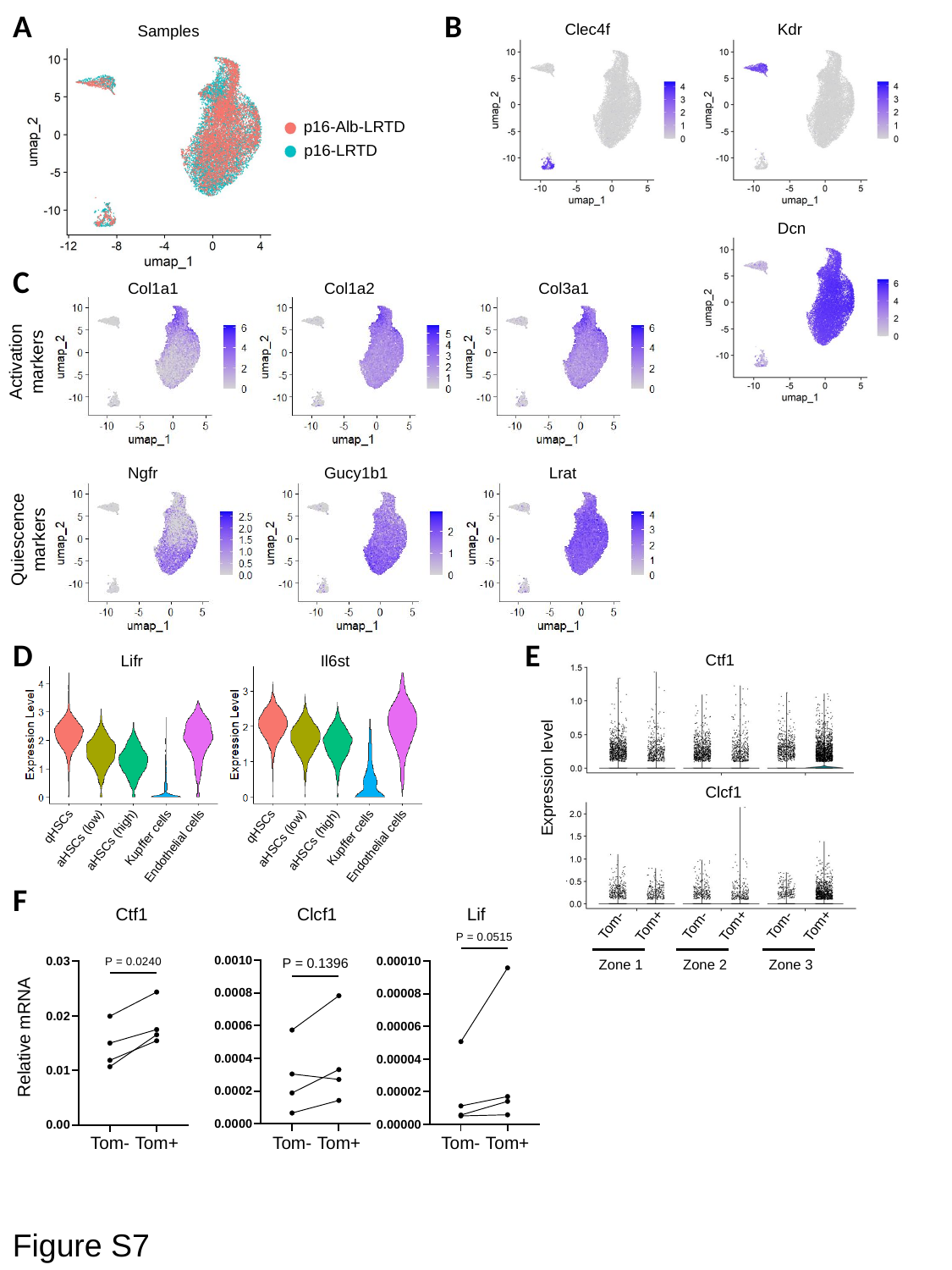

A
B
Clec4f
Kdr
Dcn
Samples
p16-Alb-LRTD
p16-LRTD
C
Col1a1
Col1a2
Col3a1
Activation markers
Ngfr
Gucy1b1
Lrat
Quiescence markers
D
E
Ctf1
Expression level
Clcf1
Tom-
Tom+
Tom-
Tom+
Tom-
Tom+
Zone 1
Zone 2
Zone 3
Lifr
Il6st
qHSCs
qHSCs
Kupffer cells
Kupffer cells
aHSCs (low)
aHSCs (low)
aHSCs (high)
aHSCs (high)
Endothelial cells
Endothelial cells
F
Ctf1
Clcf1
Lif
Relative mRNA
Tom-
Tom+
Tom-
Tom+
Tom-
Tom+
Figure S7

## Slide 8
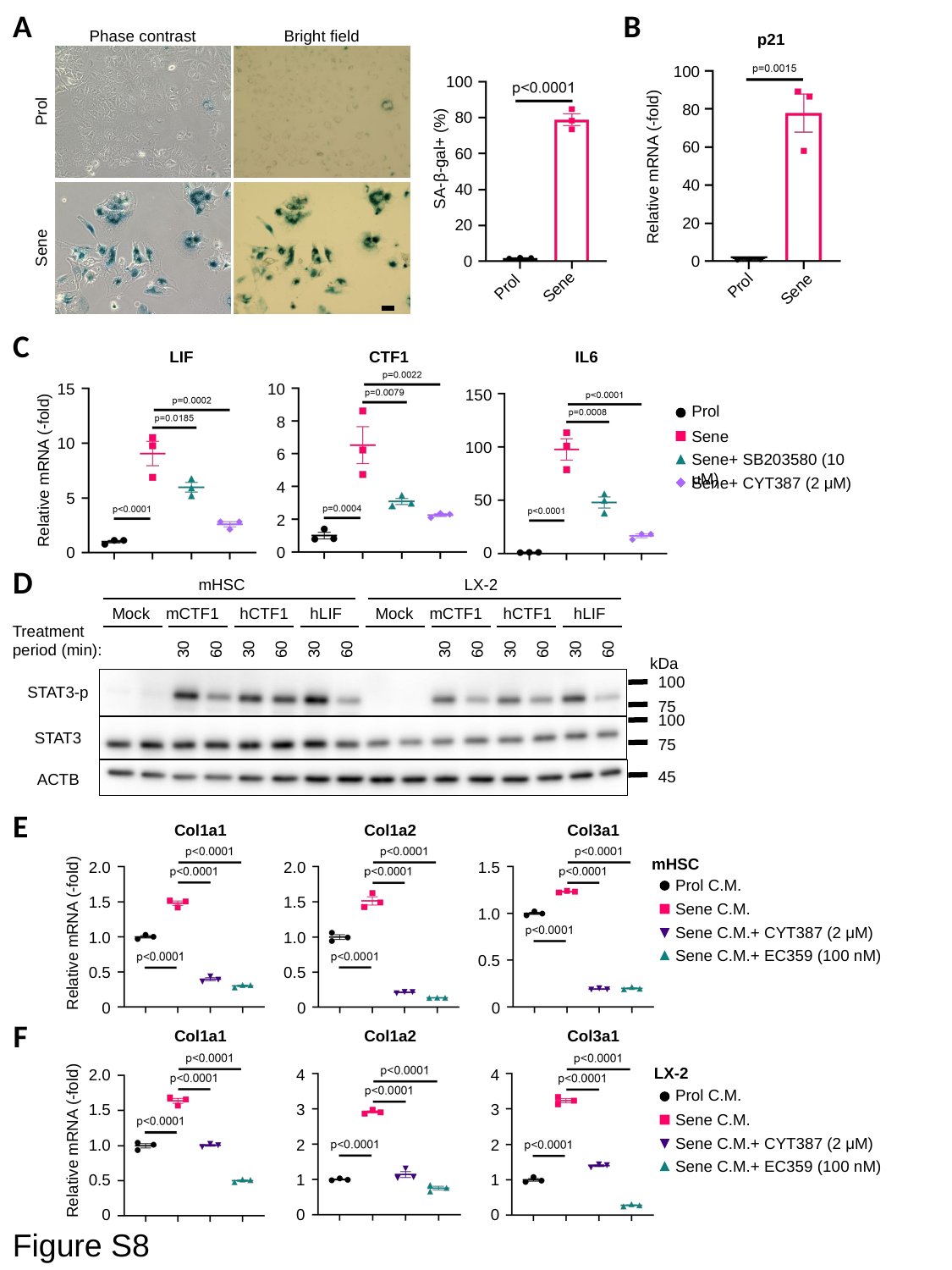

A
B
Phase contrast
Bright field
Prol
Sene
p21
100
80
60
Relative mRNA (-fold)
40
20
0
Prol
Sene
100
80
60
SA-β-gal+ (%)
40
20
0
Sene
Prol
C
LIF
CTF1
IL6
15
10
150
Prol
8
Sene
10
100
6
Sene+ SB203580 (10 μM)
Relative mRNA (-fold)
Sene+ CYT387 (2 μM)
4
5
50
2
0
0
0
D
mHSC
LX-2
Mock
mCTF1
hCTF1
hLIF
Mock
mCTF1
hCTF1
hLIF
Treatment
period (min):
30
60
30
60
30
60
30
60
30
60
30
60
kDa
100
STAT3-p
75
100
STAT3
75
45
ACTB
E
Col1a1
Col1a2
Col3a1
mHSC
2.0
2.0
1.5
Prol C.M.
1.5
1.5
Sene C.M.
1.0
Sene C.M.+ CYT387 (2 μM)
Relative mRNA (-fold)
1.0
1.0
Sene C.M.+ EC359 (100 nM)
0.5
0.5
0.5
0
0
0
F
Col1a1
Col1a2
Col3a1
LX-2
2.0
4
4
Prol C.M.
1.5
3
3
Sene C.M.
Relative mRNA (-fold)
Sene C.M.+ CYT387 (2 μM)
1.0
2
2
Sene C.M.+ EC359 (100 nM)
0.5
1
1
0
0
0
Figure S8

## Slide 9
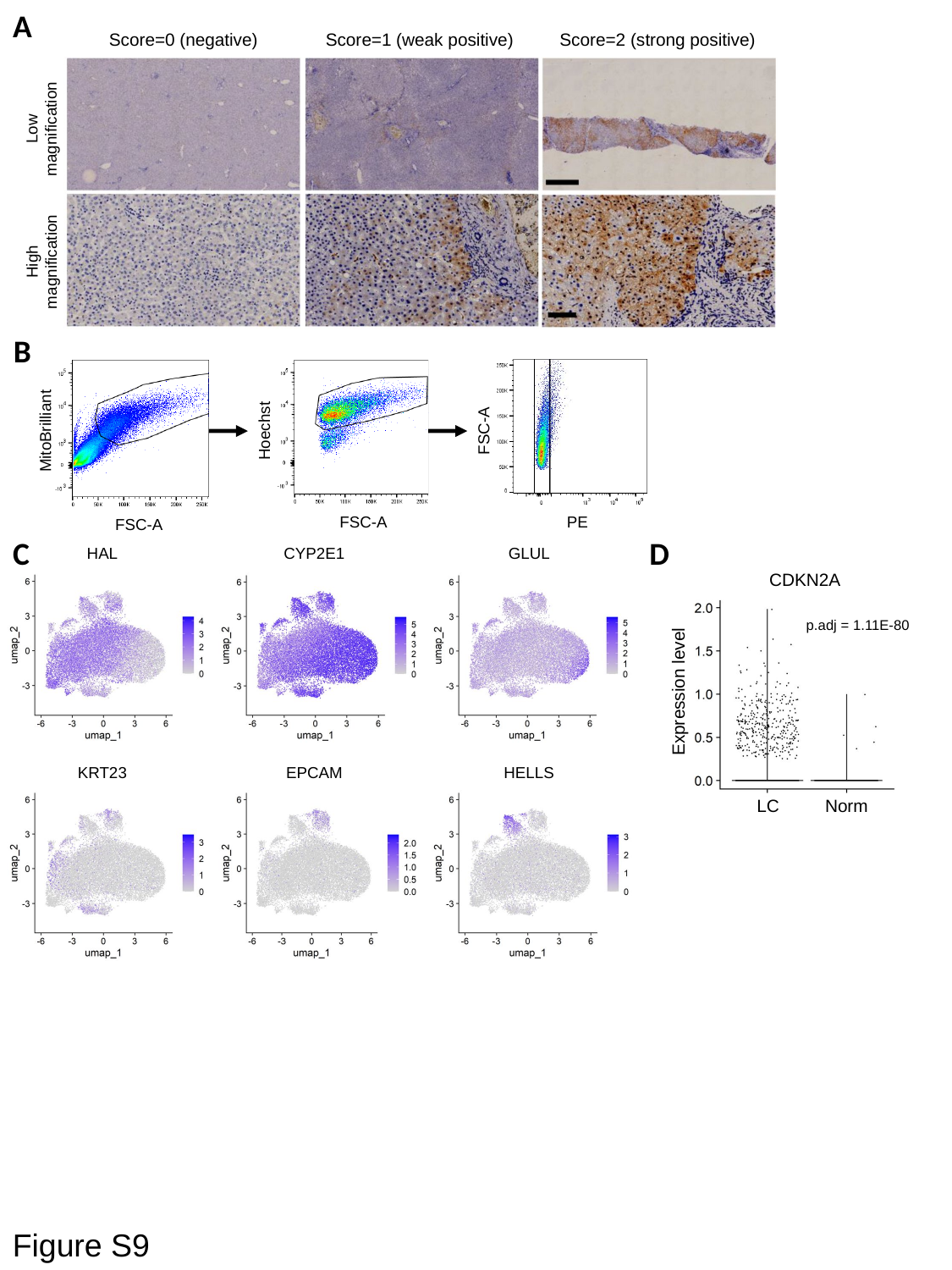

A
Score=0 (negative)
Score=1 (weak positive)
Score=2 (strong positive)
Low magnification
High magnification
B
MitoBrilliant
Hoechst
FSC-A
FSC-A
PE
FSC-A
C
D
HAL
CYP2E1
GLUL
CDKN2A
p.adj = 1.11E-80
Expression level
KRT23
EPCAM
HELLS
LC
Norm
Figure S9

## Slide 10
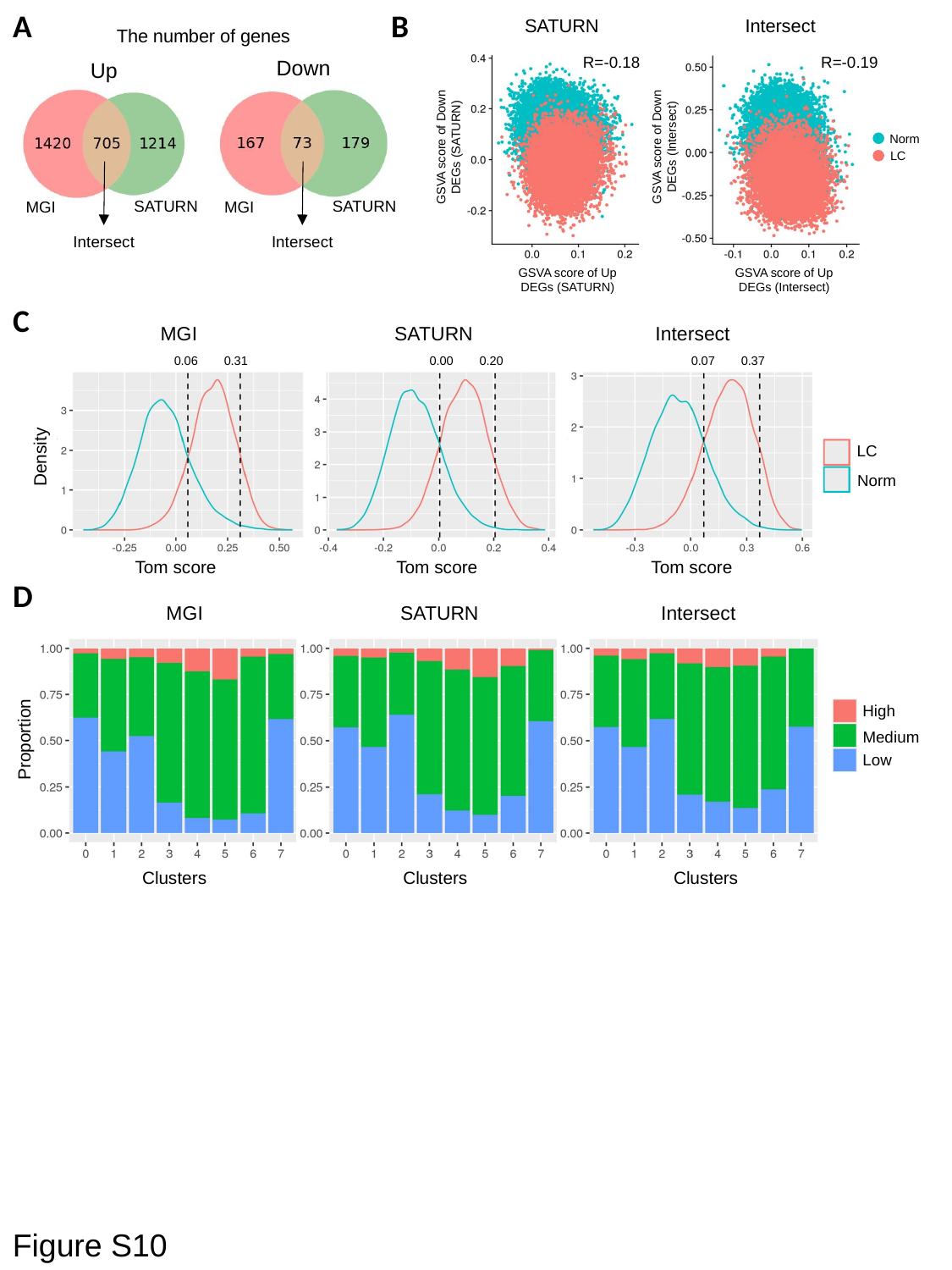

A
B
SATURN
Intersect
R=-0.18
R=-0.19
Norm
GSVA score of Down DEGs (Intersect)
GSVA score of Down DEGs (SATURN)
LC
GSVA score of Up DEGs (SATURN)
GSVA score of Up DEGs (Intersect)
The number of genes
Down
Up
SATURN
SATURN
MGI
MGI
Intersect
Intersect
C
MGI
SATURN
Intersect
0.06
0.31
0.00
0.20
0.07
0.37
LC
Density
Norm
Tom score
Tom score
Tom score
D
MGI
SATURN
Intersect
High
Medium
Proportion
Low
Clusters
Clusters
Clusters
Figure S10

## Slide 11
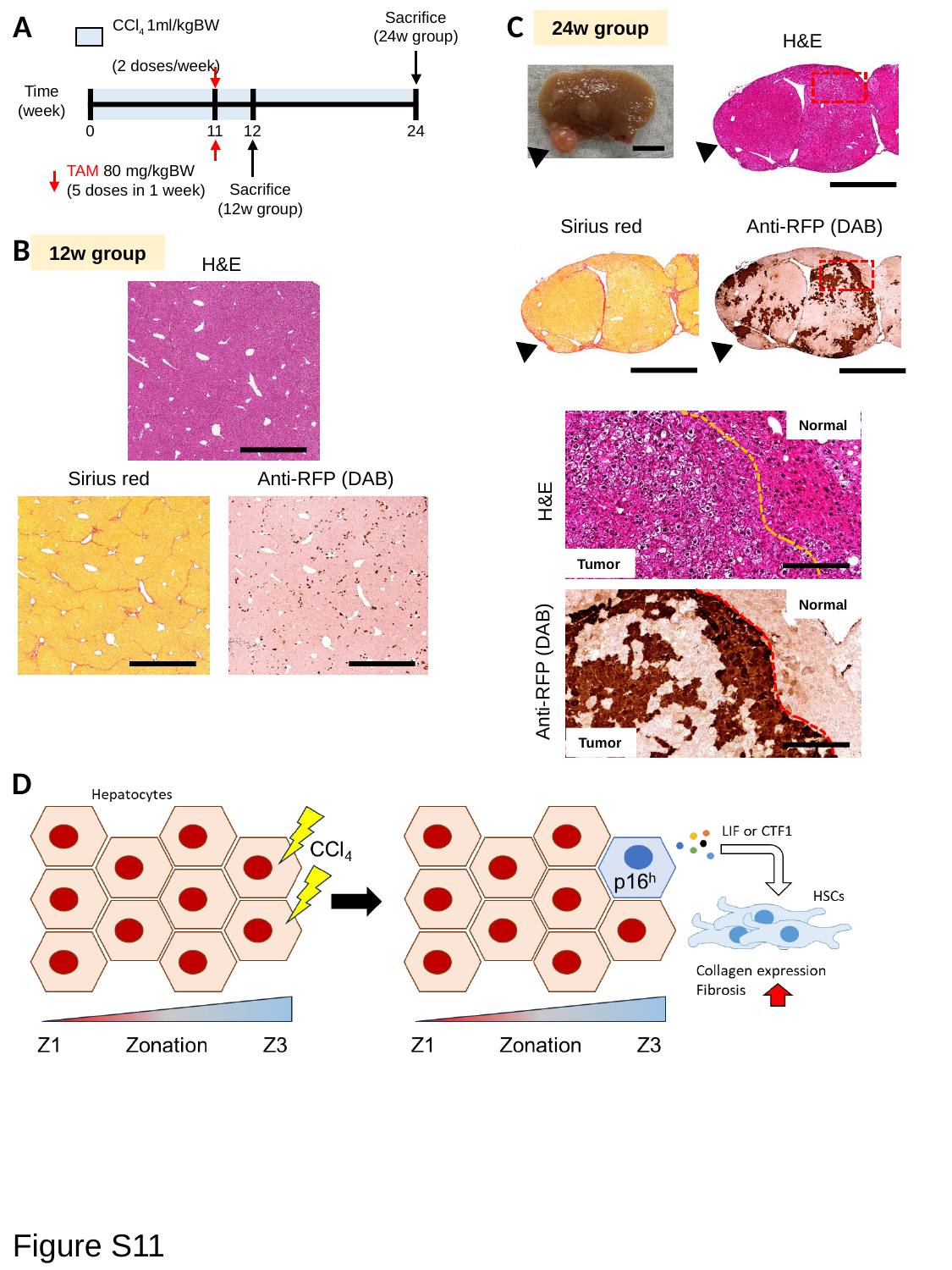

A
C
Sacrifice
(24w group)
CCl4 1ml/kgBW
(2 doses/week)
24w group
H&E
Sirius red
Anti-RFP (DAB)
Time
(week)
0
11
12
24
TAM 80 mg/kgBW
(5 doses in 1 week)
Sacrifice
(12w group)
B
12w group
H&E
Sirius red
Anti-RFP (DAB)
Normal
H&E
Tumor
Normal
Anti-RFP (DAB)
Tumor
D
Figure S11
