# Supplementary material for: p16Ink4a‐Positive Hepatocytes Drive Liver Fibrosis Through Activation of LIFR Family Pathway
Source: Adv Sci (Weinh). 2026 Jan 25;13(17):e10562. doi: 10.1002/advs.202510562 (PMC13042423; doi:10.1002/advs.202510562)
Supplement: Supplementary file 3 — Supporting File 3: advs73862‐sup‐0003‐TableS2.docx. [file ADVS-13-e10562-s001.docx]

**Table S1 Sequences of qPCR primers**

| **Genes** | **F/R** | **Sequences** |
| --- | --- | --- |
| mGapdh | F | GCCTTCCGTGTTCCTACCCC |
|  | R | GCCTGCTTCACCACCTTCTTG |
| mp16^Ink4a^ | F | GAACTCTTTCGGTCGTACCC |
|  | R | AGTTCGAATCTGCACCGTAGT |
| tdTomato | F | CTTTCCGGGACTTTCGCTTTCC |
|  | R | AACACCACGGAATTGTCAGTGC |
| Cre | F | AACGAGTGATGAGGTTCGCA |
|  | R | AGGTTCTGCGGGAAACCATT |
| mCol1a1 | F | TAGGCCATTGTGTATGCAGC |
|  | R | ACATGTTCAGCTTTGTGGACC |
| mCol1a2 | F | GGTGAGCCTGGTCAAACGG |
|  | R | ACTGTGTCCTTTCACGCCTTT |
| mCol3a1 | F | GCCCACAGCCTTCTACACCT |
|  | R | GCCAGGGTCACCATTTCTC |
| mActa2 | F | TCCTGACGCTGAAGTATCCGATA |
|  | R | GGTGCCAGATCTTTTCCATGTC |
| mGpx3 | F | ATTTGGCTTGGTCATTCTGG |
|  | R | CCACCTGGTCGAACATACTTG |
| mNqo1 | F | AGGATGGGAGGTACTCGAATC |
|  | R | AGGCGTCCTTCCTTATATGCTA |
| mWnt5a | F | CTCCTTCGCCCAGGTTGTTATAG |
|  | R | TGTCTTCGCACCTTCTCCAATG |
| mWee1 | F | AAGAGCGCAGAGCAGTTACG |
|  | R | GCTGCCATTTGGGCTTTCTTG |
| mAnxa2 | F | CACCAACTTCGATGCTGAGAGG |
|  | R | GCACATTGCTGCGGTTTGTCAG |
| mCd63 | F | GGAAGAACATACTGCTGGTGGC |
|  | R | ACAGCAGGAGAAGATAATTCCCAAG |
| mp57 | F | GGGCAGTACAGGAACCATTTC |
|  | R | TTAGCTTACAGTGTCCCGCA |
| mGstm1 | F | CCAAACCTGAGGGACTTCCTGG |
|  | R | TAGTGAGTGCCCGTGTAGCAAG |
| mGstm3 | F | CAAACCTGAGGGACTTCCTGGC |
|  | R | CTGAGTGACAGCATGGCAGGG |
| mGsta3 | F | GGTGCAGACCAAAGCCATTCTC |
|  | R | GGGGCATGTGGGGGTAATAGAG |
| mTxnip | F | ATGTACGCCCCTGAGTTCCAGTTC |
|  | R | GATGCTTCATTTCCTGCAGGCTCAC |
| mCtf1 | F | CATTTGGAGGCCAAGATCCGC |
|  | R | AAAGGGCTCTCCCTGTTGCTG |
| mClcf1 | F | CTTAGCTGGGACCTACCTGAA |
|  | R | CCACACTTCCAAGTTGACCGT |
| hGAPDH | F | AAGGTGAAGGTCGGAGTCAA |
|  | R | AATGAAGGGGTCATTGATGG |
| hACTA2 | F | CAGCCAAGCACTGTCAGG |
|  | R | CCAGAGCCATTGTCACACAC |
| hCOL1A1 | F | AAGAGGAAGGCCAAGTCGAG |
|  | R | CACACGTCTCGGTCATGGTA |
| hCOL1A2 | F | GGCCCTCAAGGTTTCCAAGG |
|  | R | CACCCTGTGGTCCAACAACTC |
| hCOL3A1 | F | AGGGGAGCTGGCTACTTCTC |
|  | R | AGGACTGACCAAGATGGGAA |
